# Removal of Bisphenol A, Bisphenol S, and Estrogenic Activity from Real Wastewater Using a Multi-stage IFAS System

**DOI:** 10.1007/s00128-026-04204-3

**Published:** 2026-03-15

**Authors:** Amanda F. do Amaral, Alexandre S. A. da Silva, Deivisson L. Cunha, Priscila M. de O. M. Cunha, Rodrigo Coutinho, Marcia Marques

**Affiliations:** Department of Sanitary and Environmental Engineering, Rio de Janeiro State University (UERJ), São Francisco Xavier, 524, Rio de Janeiro, RJ CEP 20550-900 Brazil

**Keywords:** Bisphenol A, Bisphenol S, Endocrine-disrupting compounds, Integrated fixed-film activated sludge, Wastewater treatment, Yeast estrogen screen

## Abstract

This study evaluated the removal of bisphenol A (BPA), bisphenol S (BPS), and estrogenic activity from real municipal wastewater using a pilot-scale multi-stage integrated fixed-film activated sludge (MS-IFAS) system. The configuration comprised five sequential reactors (Anaerobic, Anoxic-1, Aerobic under IFAS, Anoxic-2, and Re-aeration) providing distinct redox conditions. Influent concentrations averaged 112.6 ± 49.5 µg L^−1^ for BPA and 6.6 ± 3.0 µg L^−1^ for BPS. Overall removal efficiencies reached 99.8 ± 0.1% and 97.7 ± 1.0%, respectively, despite influent variability. The aerobic IFAS reactor was the main contributor to contaminant degradation, maintaining performance even after a > 90% biomass loss. Estrogenicity, assessed by the Yeast Estrogen Screen assay and expressed as 17β-estradiol equivalents (E2-EQ) decreased by 83.2% (0.031–0.005 ng L^−1^). Effluent E2-EQ values were below ecotoxicological thresholds, indicating negligible estrogenic risk to aquatic organisms. These highlight the strong potential of MS-IFAS for removal of xenoestrogenic contaminants in real wastewater.

**Supplementary Information:**

The online version contains supplementary material available at 10.1007/s00128-026-04204-3.

## Introduction

Over recent decades, synthetic organic compounds with endocrine-disrupting effects, particularly bisphenols, have raised global concern due to their persistence and widespread occurrence in aquatic environments (Puri et al. 2023). Bisphenol A (BPA), historically the most extensively studied endocrine disrupting chemical (Rubin 2011), is still detected in wastewater treatment plants (WWTPs) worldwide at concentrations ranging from a few ng L^−1^ to hundreds of µg L^−1^ (Xue and Kannan 2019; Petrie et al. 2019; Qian et al. 2021; Grobin et al. 2024; Puri et al. 2024), with exceptionally high levels reported in Brazil (Coutinho et al. 2022). Besides estrogenic effect, BPA presents several signaling pathways regarded as non-estrogenic modes of action (Yuan et al. 2023).

Regulatory restrictions have stimulated the replacement of BPA by structural analogs, mainly bisphenol S (BPS) (Loganathan et al. 2023). However, evidence indicates that BPS can induce endocrine-disrupting effects, with studies reporting endocrine-related responses in fish models (Berto-Júnior et al. 2018; Frenzilli et al. 2021; Qiu et al. 2021).

Despite numerous reports on the occurrence and toxicity of bisphenols, their removal in full-scale WWTPs remains highly variable and often incomplete (Nie et al. 2012; Guerra et al. 2015; Xue and Kannan 2019). In response, international regulations, such as the recent European Directive (EU) 2024/3019, have emphasized the need for advanced treatment processes targeting contaminants of emerging concern (CECs), including bisphenols (European Union 2024). A relevant increase in costs associated with wastewater treatment is expected. Therefore, improving the removal rate of these CECs in existing and new biological treatment plants to achieve higher removal efficiencies of CECs is likely the most cost-effective approach.

Among biological approaches, hybrid systems integrating suspended and fixed biomass, such as the Integrated Fixed-Film Activated Sludge (IFAS), have shown enhanced removal of organic matter, nutrients, and more recently CECs, positioning this technology as a promising alternative for upgrading conventional WWTPs (Kim et al. 2009; Waqas et al. 2020). The superior performance of IFAS compared to conventional biological systems arises from biofilm development on carriers, which increases biomass retention, supports slow-growing microbial populations, and promotes stratified redox microenvironments conducive to the degradation of recalcitrant compounds (Kim et al. 2009; Shreve and Brennan 2019). Studies have demonstrated superior removal of several CECs—including BPA—in biofilm-based systems compared to suspended biomass alone (Falås et al. 2013; Shreve and Brennan 2019). However, no reports of BPS removal in such systems were found.

A promising approach to improve CECs removal efficiency in hybrid systems, such as IFAS, is the combination of different redox conditions (Dubey et al. 2022). In this context, multi-stage IFAS (MS-IFAS) system operated under sequential anaerobic, anoxic, and aerobic conditions offer a promising configuration for enhancing CECs removal. Moreover, combining quantification of targeted xenoestrogens such as BPA and BPS by chromatography-mass spectrometry with bioassays such as the Yeast Estrogen Screen (YES) provides a robust framework for evaluating both contaminants removal and residual estrogenic activity (Routledge and Sumpter 1996).

Thus, the purpose of this research was to address this knowledge gap by investigating the removal of BPA, BPS, and overall estrogenicity in a pilot-scale MS-IFAS system fed with real municipal wastewater arriving in Alegria WWTP (Rio de Janeiro, Brazil), one of the largest in the world. To our knowledge, this is the first study to evaluate the performance of a hybrid multi-stage biological system in treating both BPA and BPS, while simultaneously quantifying residual estrogenic activity in real complex wastewater matrices.

## Materials and Methods

### Multi-Stage IFAS System (MS-IFAS)

A pilot-scale MS-IFAS system was developed based on a modified Bardenpho configuration, integrating IFAS technology to enhance biological treatment performance. The system was designed, built, and implemented at the Alegria Municipal WWTP (Rio de Janeiro, Brazil), with a treatment capacity of 2.5 m^3^ s^−1^. The influent used in the MS-IFAS system was collected downstream of the screening process and before primary treatment. This WWTP receives a complex wastewater including residential, industrial, and hospital effluents, stormwater runoff, landfill leachate, and septic tank sludge delivered by tanker trucks. Such variability in influent composition represents a major challenge for biological wastewater treatment.

**Fig. 1:**
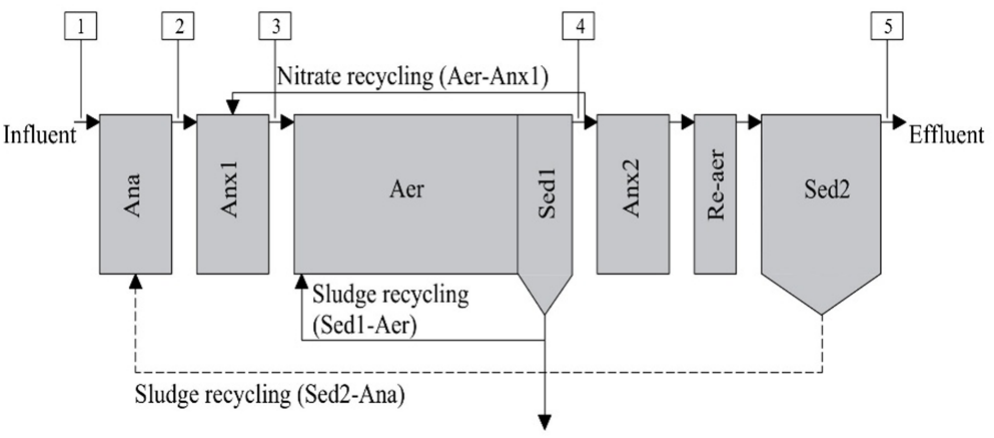
Diagram of the MS-IFAS with the following treatment units: Anaerobic reactor (Ana); Anoxic-1 reactor (Anx1); Aerobic reactor (Aer); Anoxic-2 reactor (Anx2); Re-aerated reactor (Re-aer); Sedimentation-1 tank (Sed1); Sedimentation-2 tank (Sed2). Sampling points: 1, 2, 3, 4, and 5

The MS-IFAS comprised five sequential biological reactors: Anaerobic reactor, Anoxic-1 reactor, Aerobic reactor (under IFAS configuration), Anoxic-2 reactor, and Re-aeration reactor (Fig. 1). Only the aerobic reactor employed hybrid biomass technology, including both suspended and attached microbial communities. Approximately 50% of the aerobic reactor volume was occupied by high-density polyethylene (HDPE) carriers (Enviromex; white cylinders, 26 mm outer diameter, 0.96 g cm^−3^ density, 500 m^2^ m^−3^ surface area). In order to reduce costs and operational complexity, the use of carriers was limited to the aerobic reactor.

Internal recirculation ratios were set at 1:1 from the aerobic reactor to the first anoxic reactor (Aer→Anx1) and 1:1 from the secondary sedimentation to the anaerobic reactor (Sed2→Ana) (Table S1). The 1:1 ratio were adopted as conservative baseline values for pilot operation under highly variable real wastewater, and no optimization of the recirculation was conducted in this study. Sludge returned from the first sedimentation tank to the aerobic compartment, and it was maintained at a 4:1 ratio to ensure sufficient aerobic biomass concentration for effective COD (chemical oxygen demand) oxidation and stable biofilm development.

The influent volumetric rate was initially set at 0.25 L min^−1^ and progressively adjusted throughout the operation phase. Detailed operational parameters (Tables S1 and S2), equipment specifications, flow rate variations, and procedures of physicochemical and chromatographic analyses are provided in the Supplementary Material.

During this operational period, an accidental washout event occurred, resulting in the loss of more than 90% of the suspended biomass in the MS-IFAS system. No changes were made to the operational parameters following this event, and system performance was continuously monitored before and after the washout.

### Monitoring Program

The MS-IFAS system was operated for eight months. During the first five months, efforts focused on acclimation and monitoring of key physicochemical parameters. In the last 56 days, eight weekly sampling campaigns were conducted to asses both physicochemical parameters and bisphenol concentrations were assessed (BPA and BPS). Wastewater samples were collected at five specific locations along the treatment train: (1) influent and effluents from (2) the anaerobic reactor, (3) the anoxic-1 reactor, (4) the aerobic reactor, and (5) the final treatment stage (Fig. 1).

To determine the removal efficiency of each reactor, concentrations measured at its influent and effluent were compared. Cumulative removal efficiency for the overall MS-IFAS system was calculated using the influent concentration of the raw wastewater and the effluent concentration of the final reactor. Detailed calculation procedures are provided in the Supplementary Material.

### Yeast Estrogen Screen (YES)

Estrogenicity in influent and effluent wastewater from the pilot-scale MS-IFAS system was evaluated using the Yeast Estrogen Screen (YES) assay (Routledge and Sumpter 1996). This bioassay employs a recombinant *Saccharomyces cerevisiae* strain (BJ1991) (Bistan et al. 2012) and it has been frequently applied to assess the removal of estrogenicity in combination with estrogen hormones degradation (Pastre et al. 2024). The recombinant yeast strain was incubated in 10 mL of nutrient medium at 28 °C for 24 h under agitation at 100 rpm. For assay preparation, 25 mL of fresh culture medium was supplemented with 250 μL of chlorophenol red-β-D-galactopyranoside (CPRG, 10 mg mL^−1^) and 25 μL of the yeast culture (Table S5). Wastewater samples were serially diluted in methanol and tested in duplicate. Then, 10 L of the samples and the standard were transferred to the wells of a 96-well plate. After solvent evaporation, 200 L assay medium was introduced into each well.

A calibration curve was generated using 17β-estradiol across a concentration range of 1.33 to 2724 ng L^−1^, with methanol used as the solvent control. The microplates were agitated for 2 min and then incubated at 28 °C. After a 72-h incubation period, absorbance readings were taken at 575 and 620 nm with a SpectraMax microplate reader. Estrogenic activity was quantified as estradiol-equivalent concentrations (EQ-E2, ng L^−1^).

### Data Analysis

The Eq. 1 below was applied to correct the measured absorbance values, thereby removing the interference of turbidity on the estrogenic response:

1 $$ Abs_{{cor\left( {sample} \right)}} = Abs_{{575\left( {sample} \right)}} - Abs_{{620\left( {blanks} \right)}} $$

Values were subsequently plotted, and the resulting response curves exhibited a sigmoidal pattern. Data fitting was performed using a symmetrical logistic function with the software Origin 2020 (OriginLab). The 17β-estradiol equivalents (E2-EQ), expressed in ng L^−1^, was determined by interpolating the experimental results into the standard E2 dose–response curve (Fig. S2) using a log-logistic model (Eq. 2):

2 $$ y = \left( {\frac{A1 - A2}{{1 + \left( \frac{x0}{x} \right)^{p} }} + A2} \right) $$

Parameters A₁ and A₂ represent the maximum and minimum induction responses of β-galactosidase, respectively, expressed as corrected absorbance values. The parameter x₀ corresponds to the median effective concentration (EC50%) of E2, in ng L-1, while p describes the slope of the sigmoidal curve. The ordered pair (x, y) refers to the concentration of the analyzed sample and its corresponding corrected absorbance response. Finally, the E2-EQ was estimated as the lowest x value capable of eliciting an agonistic response, normalized by the final sample enrichment factor applied in the assay (Argolo et al. 2021).

### Statistical Analysis

All statistical analyses were performed in R (*p* < 0.05). Data normality and homogeneity of variances were assessed using the Shapiro–Wilk and Breusch–Pagan tests, respectively, to guide the selection of subsequent statistical methods. Differences among reactors were tested using the non-parametric Kruskal–Wallis test (Table S6), and Dunn’s post hoc test was employed to determine which specific groups differed from each other (Table S7). Bartlett’s test of sphericity (*p* < 0.05) confirmed sufficient intercorrelation among variables, validating the application of principal component analysis (PCA) (Table S8). The results of the PCA are presented in Tables S9 and S10. Correlations were assessed by Pearson or Spearman tests depending on data distribution (Table S11). To compare paired removals of BPA and BPS within reactors, independent-samples t-tests were used for normally distributed data and Mann–Whitney tests when normality was not met (Table S12). The Mann–Whitney test was also used to compare the influent versus effluent estrogenicity measured by the YES assay.

## Results and Discussion

### BPA and BPS Occurrence in Wastewater and Removal by the MS-IFAS

During 56 (fifty-six) days of monitoring, BPA and BPS were consistently detected at high concentrations in the influent of the MS-IFAS system collected after screening and prior to primary treatment at a full-scale WWTP. BPA was the predominant substance identified in the influent, with levels varying between 57,000 and 181,000 ng L^−1^ and a mean concentration of 113,000 ± 49,500 ng L^−1^. These levels are markedly elevated in comparison to those documented in influents of WWTPs across various countries, such as: China (271.8 to 602.7 ng L^−1^), United States (50.3 to 74.4 ng L^−1^), United Kingdom (874 ± 266 to 37,200 ± 31,700 ng L^−1^), India (79.1 ± 9.34 ng L^−1^) and, Slovenia (2,379 ng L^−1^) (Petrie et al. 2019; Xue and Kannan 2019; Qian et al. 2021; Grobin et al. 2024; Puri et al. 2024). Comparable concentrations, however, have been reported in Brazil, with values of 84,110 ± 4,200 ng L^−1^ and 62,010 ± 3,100 ng L^−1^ in two WWTPs treating mixed domestic and industrial wastewater in Curitiba (Froehner et al. 2011), and up to 140,260 ng L^−1^ in a smaller WWTP in Ceará (Vidal et al. 2020).

The elevated BPA levels observed in this study are likely to be due to the co-treatment of landfill leachate at the WWTP, a common practice in Brazil (Pereira et al. 2023). Previous reports indicate that BPA concentrations may exceed 5 mg L^−1^ in leachate (Ferrer-Polonio et al. 2021).

As anticipated, BPS was detected at lower concentrations, ranging from 1,684.0 to 11,216.9 ng L^−1^. These values surpass those reported for untreated wastewater in various countries, including China (73.4–152.9 ng L^−1^), Slovenia (3,009 ng L^−1^), and Romania (47.9–1,688 ng L^−1^) (Chiriac et al. 2020; Qian et al. 2021; Grobin et al. 2024).

Despite the elevated concentrations of target contaminants in the highly variable influent received by the pilot-scale MS-IFAS system (Table S2), the effluent exhibited substantially reduced levels. Effluent concentrations ranged from 106.1 to 384.7 ng L^−1^ for BPA and from 72.3 to 356.6 ng L^−1^ for BPS. During the monitoring period, the MS-IFAS demonstrated removal efficiency greater than 99.5% for BPA and 95.7% for BPS. These removal rates were significantly higher than those reported for two full-scale conventional WWTPs using activated sludge technology, where average BPA removal efficiencies were 34% and 52%, while BPS removal was either negligible or negative, with a maximum of 1.1% (Xue and Kannan 2019). In contrast, full-scale wastewater treatment plants operating under different redox conditions have achieved BPA and BPS removal efficiencies greater than 90% (Nie et al. 2012; Shreve and Brennan 2019; Gu et al. 2021). Notably, these studies did not evaluate the individual contribution of different redox conditions, an important aspect requiring further investigation.

### Performance of Each Compartment of MS-IFAS

**Fig. 2:**
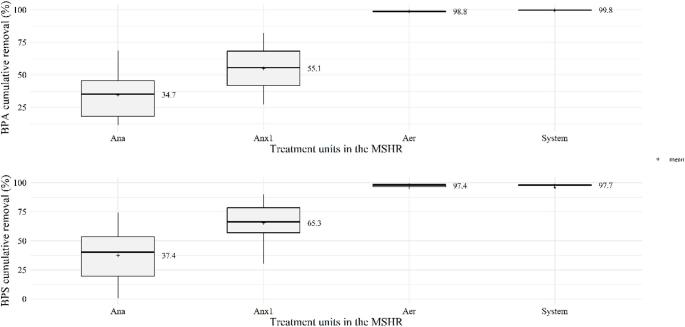
**a** BPA and **b** BPS removal from wastewater by each reactor (Anaerobic, Anoxic-1, Aerobic) and by the entire MS-IFAS system

BPA removal reached 34.7 ± 20.6% in the anaerobic reactor and increased to 55.1 ± 20.2% after the anoxic-1 reactor. Nevertheless, the difference between the individual removal efficiencies was not statistically significant (*p*-value = 0.925; Table S7. In contrast, the aerobic reactor played a critical role in BPA removal, achieving an average efficiency of 97.3 ± 1.5%, which was significantly higher than the removals observed in the anaerobic and anoxic-1 reactors (*p*-value = 0.037 and 0.036, respectively). After this stage, BPA removal reached 98.8 ± 0.8%, and the final system efficiency was 99.8 ± 0.1% (Fig. 2). For BPS, cumulative removal efficiencies increased progressively, reaching 37.4 ± 20.6% after the anaerobic reactor, 65.3 ± 20.4% after the anoxic-1 reactor, and 97.4 ± 1.8% after the aerobic reactor. However, when comparing the individual removal efficiencies of these reactors, the aerobic stage performed significantly better than the anaerobic and anoxic-1 reactors (*p* = 0.020 and 0.033, respectively). Total BPS removal reached 97.7 ± 1.0% (Fig. 2). It was concluded that both anaerobic and anoxic processes played significant roles in removing BPA and BPS, although the removal rates in these stages were more variable compared to the stable, consistent, and superior removal rate by the aerobic reactor (Fig. 2).

**Fig. 3:**
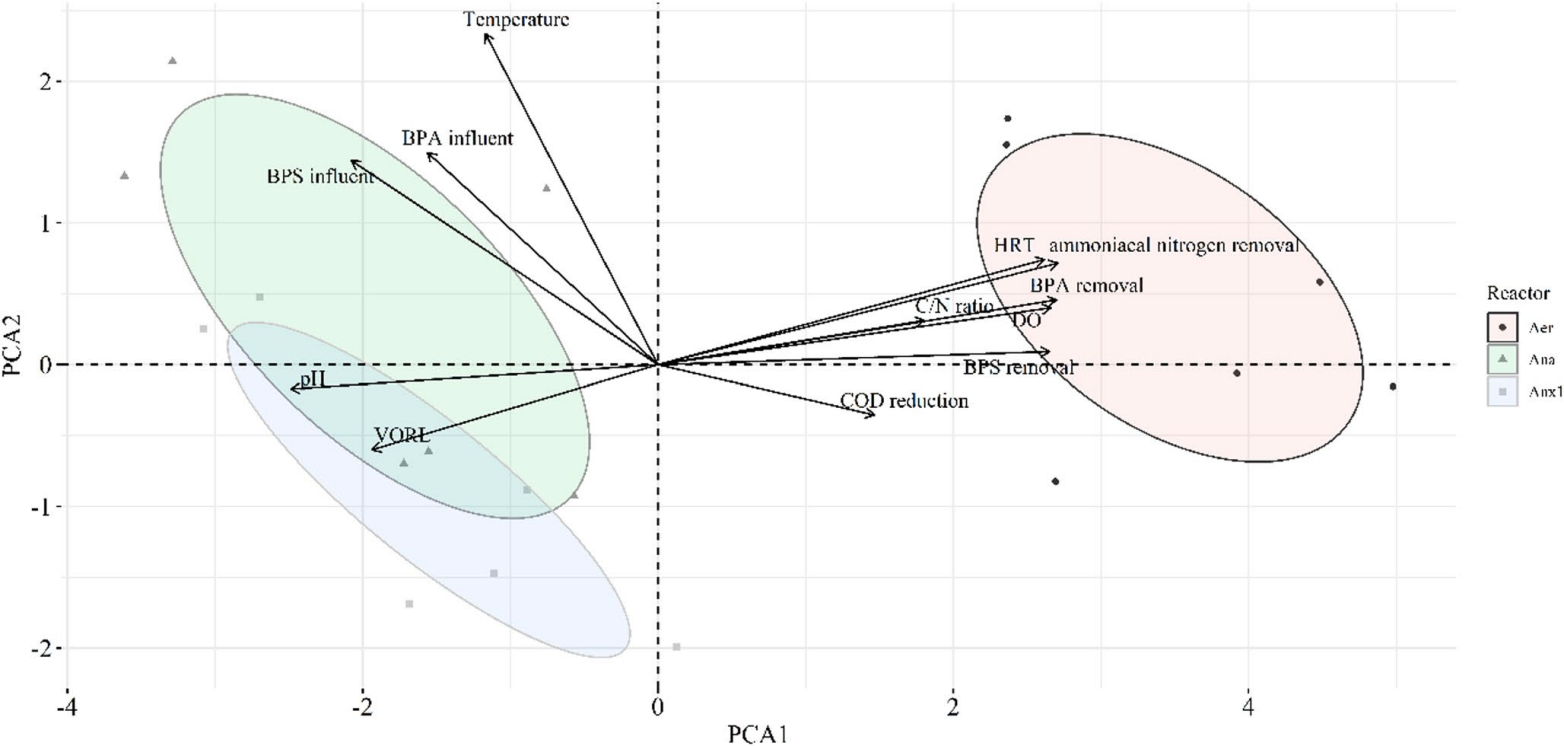
PCA including operating conditions and removal efficiency obtained by three reactors. Variables: DO, temperature, pH, VOLR (volumetric organic rate loading), C/N ratio, COD reduction, HRT, ammoniacal nitrogen removal, and influent BPA and BPS concentrations

Beyond redox conditions, several operational parameters can also influence the dynamics of BPA and BPS removal Principal Component Analysis (PCA) was applied to identify the main operational drivers influencing contaminant removal (Fig. 3). The first two components (PC1 and PC2) explained 72.5% of the total variance, which exceeds the threshold commonly considered sufficient in environmental multivariate analyses. In the biplot (Fig. 3), the aerobic reactor formed a clearly distinct cluster from the anaerobic and anoxic-1 reactors, reflecting its contrasting operational conditions. Its position was associated with higher oxygen dissolved (DO) and hydraulic retention time (HRT) values and with greater apparent removal of BPA, BPS, and ammoniacal nitrogen, indicating the strong influence of oxygen availability and longer retention times on overall removal performance, potentially involving a combination of biotransformation and partitioning processes.

BPA and BPS removals showed statistically significant correlations with the decrease in COD levels and ammoniacal nitrogen removal (*p* < 0.05; *r* > 0.5; Table S11. These results suggest straight interaction between these processes, indicating similar degradation mechanisms, mainly related to biodegradation. These findings corroborate prior studies recognizing biodegradation as the main mechanism responsible for BPA and BPS elimination from wastewater (Sun et al. 2017; Qian et al. 2021). Negative correlations with volumetric organic loading rate (VORL; *r* ≈ –0.76; *p* < 0.001) indicated that higher organic loads may hinder bisphenol removal, possibly due to microbial competition, where the heterotrophic community, favored by higher organic loads, predominates over autotrophic microorganisms (Miao et al. 2018). Consequently, there is a possible reduction in the activity of ammonia-oxidizing bacteria (AOB), which is known to act as a critical component in the co-metabolic removal of BPA and BPS (Roh et al. 2009; Arias et al. 2018). Positive associations with the COD/ammoniacal nitrogen (C/N ratio; *r* > 0.6; *p* < 0.01) suggest that an adequate balance between carbon and nitrogen may be an important factor to optimize the removal of this CECs. The C/N ratio directly influences microbial activity by modulating both substrate availability for heterotrophic metabolism and the performance of autotrophic microorganisms (Ahmadi et al. 2023).

BPA and BPS removal efficiencies increased with longer HRT (*p* = 0.000), demonstrating a strong positive correlation (*r* = 0.906 and 0.788, respectively), indicating that longer retention times mean more time available for microbial action. Studies show that increasing HRT improves BPA removal (Buhari et al. 2023). Consistently, a study analyzing twelve full-scale WWTPs utilizing various biological treatment processes found that BPA removal efficiency was strongly influenced by HRT, with a statistically significant correlation (Guerra et al. 2015).

It should be noted that, in addition to biodegradation, partitioning of BPA and BPS onto sludge and biofilm may contribute to the observed removal in MS-IFAS. Sorption-driven removal of bisphenols has been widely reported, particularly for BPA, which exhibits moderate hydrophobicity and higher affinity for organic matter and biomass (Rogers 1996; Wang et al. 2019). Differences in sorptive behavior between BPA and BPS have been attributed to their physicochemical properties (Table S13), especially their octanol–water partition coefficients (log Kow), with BPA showing a higher sorption potential than the more hydrophilic BPS (Rogers 1996). As sludge-phase concentrations were not analyzed in the present study, the relative contributions of biodegradation and partitioning cannot be quantitatively distinguished. Therefore, the reported removal should be interpreted as overall, reflecting the combined influence of biological and physicochemical processes.

However, since each reactor in a MS-IFAS operates under different conditions, a detailed assessment of each reactor's performance is essential to elucidate the removal mechanisms of each bisphenol.

### Anaerobic Reactor

In contrast to previous studies conducted under anaerobic conditions with upflow anaerobic sludge blanket (UASB) technology, in which BPA exhibited recalcitrance (Arias et al. 2018), the anaerobic reactor in the present study achieved an average BPA removal of 34.7 ± 20.6%, ranging from 11.3% to 68.7%. This efficiency surpassed that reported for three UASB reactors, where BPA accumulation was observed, particularly at longer HRTs (Queiroz et al. 2012). The observed removal may be attributed to a combination of mechanisms, including enhanced partitioning of BPA onto the retained biomass and solids, favored by stable operational conditions (e.g., HRT, VOLR, and pH), as well as potential biotransformation processes, possibly supported by the presence of co-substrates promoting cometabolic activity.

BPS exhibited a similar trend, with an average removal of 37.4 ± 26.6%, showing no significant difference from BPA (*p*-value = 0.902). Therefore, no clear preference for the removal of either substance under anaerobic conditions was observed. However, previous studies have reported varying results. Sun et al. (2017) found that, in a WWTP employing the anaerobic/anoxic/oxic (A2/O) process, the anaerobic phase was more effective in removing BPA (56%) compared to BPS (33%). Conversely, biodegradation tests indicate that BPS degrades more rapidly under anaerobic conditions than BPA (Ike et al. 2006), highlighting the importance of evaluating operational conditions that influence the removal of these contaminants.

The removal of both bisphenols showed a strong inverse relationship with pH (*r* > 0.8; *p* < 0.05; Table S11). Variation in pH influences the behavior of CECs by affecting their protonation state, water solubility, and adsorption potential (Roman et al. 2020; Qian et al. 2021). BPA and BPS have pKa (acid dissociation constant) values of approximately 9.6 and 8.2, respectively, classifying them as polar organic compounds with higher solubility in alkaline conditions (Zeng et al. 2006). Under acidic conditions, these substances tend to shift from the aqueous to the solid phase, enhancing their adsorption potential. This observation is consistent with the statistically significant inverse correlation between pH and the removal efficiencies of BPA and BPS observed in this study.

### Anoxic-1 Reactor

The anoxic-1 reactor achieved average removal efficiencies of 33.4 ± 12.3% for BPA and 46.8 ± 16.8% for BPS, with no significant difference between the two compounds (*p* = 0.165). These rates were higher than those reported in a full-scale WWTP operating with the A2/O process, where the anoxic stage removed less than 10% of both contaminants (Sun et al. 2017), indicating enhanced performance under the present operational conditions.

The analysis revealed a highly significant correlation between HRT and BPA removal (*r* = 0.993, *p*-value = 0.001; Table S11). Previous studies suggest that increasing HRT expands the contact time between the effluent and the microbial community, thereby promoting biodegradation in WWTPs (Kumar et al. 2022), as it. In addition, ammoniacal nitrogen removal showed a linear correlation with BPS removal (*r* = 0.914, *p*-value = 0.004), consistent with the anaerobic reactor results. Nonetheless, no significant linear association was observed between denitrification and the removal of BPA or BPS, even though previous studies have demonstrated BPA degradation under denitrifying conditions (Ahmed et al. 2017). Although the anoxic-1 reactor is primarily designed for denitrification, the occurrence of partial nitrification may have contributed to bisphenol degradation (Table S2).

### Aerobic Reactor

The aerobic reactor, the only unit combining fixed and suspended biomass, achieved average BPA removal of 97.3 ± 1.5% and BPS removal of 92.2 ± 5.4%, with BPA removal significantly higher (*p* = 0.011), which is consistent with previous evidence showing that BPA is more readily biodegraded under aerobic conditions, whereas BPS shows lower biodegradability (Ike et al. 2006).

**Fig. 4:**
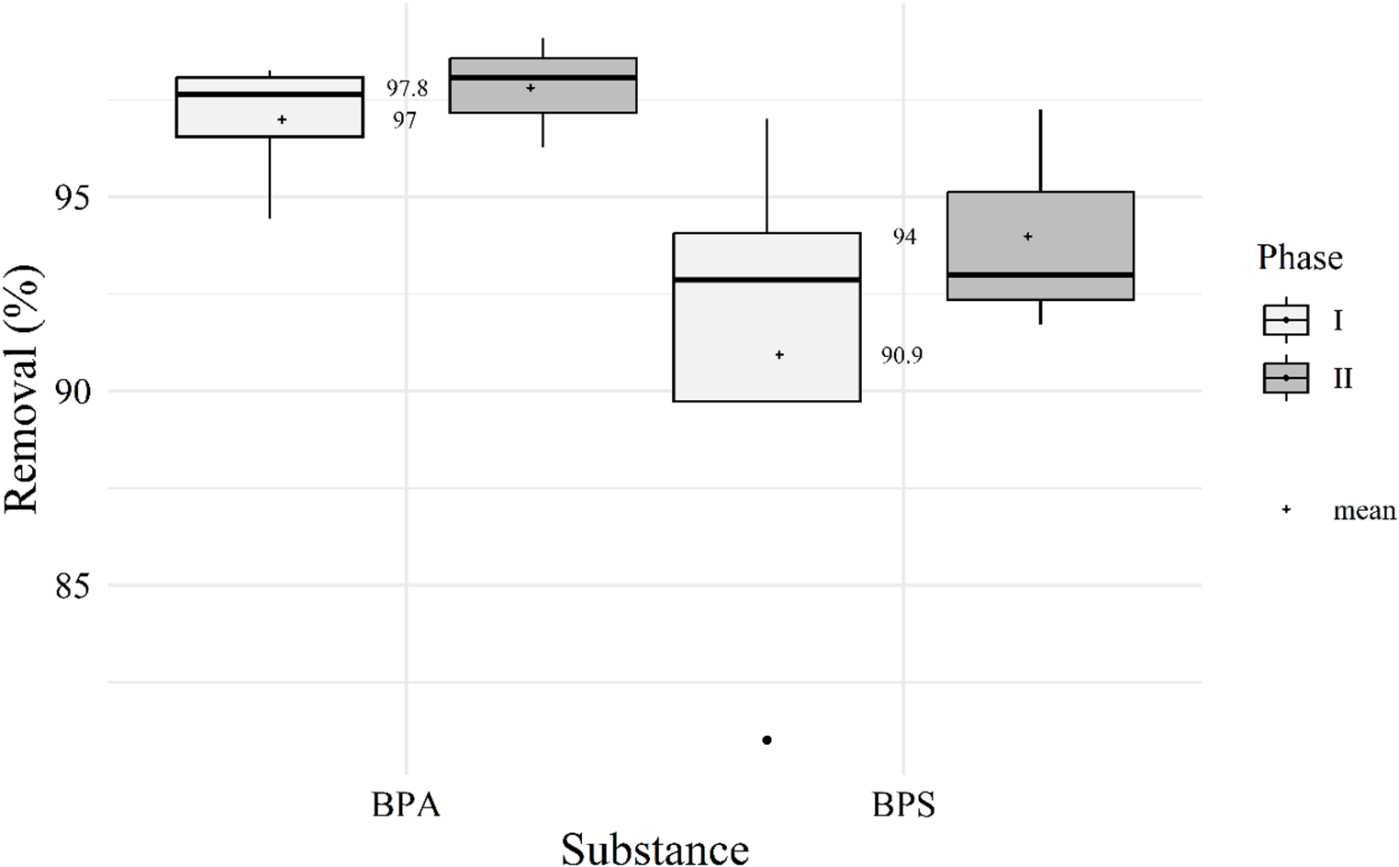
BPA and BPS removal efficiency in the aerobic reactor of the MS-IFAS system during two operational phases: Phase 1 (weeks 1 to 5) and Phase 2 (weeks 6 to 8), following an accidental washout that resulted in the loss of over 90% of the suspended biomass

On average, the aerobic reactor contained approximately 40% suspended and 60% attached biomass. Despite strong variability in influent characteristics, the MS-IFAS maintained consistently high bisphenol removal throughout operation, even after an accidental washout event that eliminated over 90% of the suspended biomass. No significant difference in BPA and BPS removal was observed before and after this event (*p* = 0.662), demonstrating the resilience of the fixed biomass and the stability of the hybrid configuration (Fig. 4).

The resilience observed in the IFAS system, where CECs removal rate remained stable despite the suspended biomass washout events has been previously reported (Arias et al. 2018). Such robustness is attributed to fixed biomass, which safeguards microbial activity under adverse conditions, retains slow-growing degraders, and enhances community diversity (Saini et al. 2023). Evidence from full-scale IFAS plants further supports this advantage, as biofilms outperformed suspended biomass in the removal of 31 CECs (Wolff et al. 2021).

BPA and BPS removals were positively correlated (*r* = 0.808; *p* = 0.028; TableSS11), suggesting that both compounds followed similar degradation dynamics in the aerobic phase. Overall, the aerobic reactor played a decisive role in bisphenol elimination, ensuring the high treatment stability observed in the MS-IFAS system.

### Estrogenicity in the Wastewater Before and After Treatment

In addition to the estrogenic activity resulting from the presence of original endocrine disrupting chemicals, biological processes may generate products that retain some estrogenic activity. An investigation supporting this interpretation showed that silico simulations of hydroxyl radical and ultraviolet-induced reactions of bisphenol analogues showing that several hydroxylated intermediates (e.g., catechol and ortho-quinone derivatives) exhibit moderate to very strong binding affinities to estrogen receptors (Porcar-Santos et al. 2024). Thus, from the environmental risk viewpoint, when bisphenols (and other endocrine disrupting chemicals eventually found in wastewater) are efficiently removed during wastewater treatment, some transformation products may still contribute to residual estrogenic activity, highlighting the importance of assessing the environmental fate and persistence of these substances. In this context, YES assay contributes with the assessment of the overall estrogenicity in both influent and effluent, regardless of the source (estrogen hormones, xenoestrogens such as bisphenols and products derived from the transformation of endocrine disruptors).

Estrogenic activity, measured as E2-EQ according to YES assays in the present investigation varied from 0.020 to 0.183 ng L^−1^ (n = 8; median value = 0.0310 ng L^−1^) in the influent of the MS-IFAS system, while in the effluent E2-EQ levels varied from 0.003 to 0.029 ng L^−1^ (n = 8; median value = 0.0055 ng L^−^1; Fig, S2; Table 1). The treatment promoted a statistically significant reduction in terms of E2-EQ levels, corresponding to an 82.3% decrease (*p* = 0.003). This demonstrates the strong capability of the MS-IFAS system to attenuate the estrogenic potential of real municipal wastewater.

**Table 1:** EQ-E2 concentrations measured in the influent (*N* = 8) and the effluent (*N* = 8) during the monitored period

	Sample	EQ-E2	*Cytotoxicity (%)	Limits of the method	
		Average (ng L^−1^)		LOD (ng L^−1^)	LOQ (ng L^−1^)
Influent	1	0.034	0%	0.01	0.02
	2	0.026	0%	0.01	0.02
	3	0.033	0%	0.01	0.02
	4	0.029	0%	0.01	0.02
	5	0.183	0%	0.01	0.02
	6	0.086	0%	0.01	0.02
	7	0.026	0%	0.01	0.02
	8	0.020	0%	0.01	0.02
Effluent	9	0.016	0%	0.01	0.02
	10	0.010	0%	0.01	0.02
	11	0.008	0%	0.01	0.02
	12	0.003	0%	0.01	0.02
	13	0.022	0%	0.01	0.02
	14	0.022	0%	0.01	0.02
	15	0.002	0%	0.01	0.02
	16	0.004	0%	0.01	0.02

*No cytotoxicity to *S. cerevisiae* was observed in any sample, even before dilution

The E2-EQ concentrations measured in the effluent (0.03 ng L^−1^ E2-EQ) were well below the bioassay trigger value of ~ 0.2 ng L^−1^ E2-EQ proposed by Conley et al. (2017) for environmentally relevant estrogenic responses in aquatic organisms. This threshold value was derived from estrogen receptor activation in the T47D-KBluc assay and can be used as a conservative screening indicator of potential ecological risk. In addition, Anderson et al. (2012) estimated predicted no-effect concentrations (PNECs), of 2 ng L^−1^ E2-EQ for chronic exposure and 5 ng L^−1^ E2-EQ for short-term exposure in U.S. surface waters. Therefore, the influent and effluent E2-EQ values observed in the present investigation are unlikely to cause endocrine disruption in aquatic organisms under typical environmental dilution scenarios.

The estrogenicity measured in the influent in this study was considered low compared to the influent of some large-scale WWTPs (Shreve and Brennan 2019; Grobin et al. 2024). Even though, it was clear that the treatment applied significantly reduced those substances causing estrogenicity in the wastewater, including those not determined in the present investigation. Previous studies have reported low estrogenic activity in urban wastewater, attributing this observation to the presence of antagonistic compounds that interfere with estrogen receptor binding, thereby diminishing the measured estrogenicity (Itzel et al. 2019). Furthermore, dissolved organic matter may interact with estrogenic compounds, forming complexes that decrease their bioavailability in the YES assay (Argolo et al. 2021). Considering that influent has much higher dissolved organic matter and possibly antagonistic substances than the effluent, the estrogenicity assessment in the effluent is likely to be more accurate than in the influent.

The estrogenicity posed by BPA and BPS is well-known (Chen et al. 2002). However, BPA has a 10^4^ to 10^5^ times lower affinity for estrogen receptors compared to 17ß-estradiol, making them much weaker endocrine disruptors (Chen et al. 2002; Gupta et al. 2024). Compared to BPA, BPS displays a reduced capacity to elicit estrogenic responses (Chen et al. 2002). Thus, despite the high concentrations of these substances quantified in wastewater, their contribution to total estrogenic activity is likely to be limited.

Kim et al. (2009) evaluated two pilot-scale treatment processes, each operating under distinct redox conditions, for processing primary wastewater sourced from a large-scale WWTP in the United States. One system consisted of aerobic reactors with IFAS technology, while the other operated exclusively with suspended biomass. The IFAS system was more efficient in removing estrogenic activity. Similarly, Shreve and Brennan (2019) evaluated six full-scale WWTPs, also operating with IFAS technology and different redox conditions, and observed removals ranging from 51.2 to 100% for estrogen receptor alpha (ERα) and from 78.9 to 98.8% for estrogen receptor beta (ERβ). These findings corroborate the present study’s evidence on the effectiveness of hybrid multi-stage systems in significantly reducing estrogenic activity in wastewater.

In summary, the MS-IFAS system not only achieved substantial statistically significant removal of bisphenols but also produced an effluent with E2-EQ concentrations well below ecotoxicologically relevant thresholds levels for aquatic organisms. The possible formation of metabolites that keep estrogenicity effect highlights the importance of applying an assay such as YES, besides analyzing the concentration reduction of endocrine disrupting chemicals to ensure a comprehensive risk assessment.

These findings suggest that, once discharged, the treated effluent is unlikely to pose endocrine disruption risks to downstream ecosystems. Nevertheless, occasional peaks observed in the influent or in the effluent because of misfunction of the biological treatment could pose episodic risks to aquatic biota in the absence of sufficient dilution or retention time in the receiving water body.

Although bisphenol concentrations and estrogenic activity were determined simultaneously, a direct correlation between individual bisphenols and E2-EQ values was not expected. The YES assay reflects the integrated estrogenic response of complex mixtures, including unidentified compounds, antagonists, and transformation products. Given the relatively low estrogen receptor affinity of BPA and BPS compared to 17β-estradiol, their contribution to overall estrogenicity is likely limited, and simple concentration–effect correlations may therefore be misleading.

## Conclusion

The efficiency of a pilot-scale MS-IFAS composed of five bioreactors (Anaerobic, Anoxic-1, Aerobic, Anoxic-2, Re-aeration) for the treatment of real municipal wastewater, with a specific focus on BPA, BPS, and estrogenicity. BPA influent concentrations of 112.6 ± 49.5 µg L^−1^ were reduced by 99.8 ± 0.1% in the effluent, while BPS influent concentrations of 6.6 ± 3.0 µg L^−1^ were reduced by 97.7 ± 1.0%. Estrogenicity measured by the YES assay was significantly reduced (*p* = 0.003) after treatment and well below the trigger value of E2-EQ for aquatic ecosystems. These findings confirm that the MS-IFAS configuration effectively removes bisphenols and reduces the estrogenic burden of the treated wastewater.

The results highlight the potential of integrating multi-stage hybrid biological systems to enhance the removal of recalcitrant estrogenic compounds without the need for tertiary or quaternary treatment steps. Future studies should extend this approach to a broader spectrum of contaminants of emerging concern to confirm its wider applicability and further consolidate the role of MS-IFAS systems as a feasible upgrading option for full-scale wastewater treatment plants.

## Supplementary Information

Supplementary Material 1
