# Supplementary material for: Removal of Bisphenol A, Bisphenol S, and Estrogenic Activity from Real Wastewater Using a Multi-stage IFAS System
Source: Bull Environ Contam Toxicol. 2026 Mar 15;116(3):67. doi: 10.1007/s00128-026-04204-3 (PMC12992361; doi:10.1007/s00128-026-04204-3)
Supplement: Supplementary file 1 — Supplementary Material 1 [file 128_2026_4204_MOESM1_ESM.docx]

**Removal of Bisphenol A, Bisphenol S, and Estrogenic Activity from Real Wastewater Using a Multi-Stage IFAS System**

Amanda F. do Amaral*; Alexandre S. A. da Silva; Deivisson L. Cunha; Priscila M. de O. M. Cunha; Rodrigo Coutinho; Marcia Marques

Department of Sanitary and Environmental Engineering, Rio de Janeiro State University (UERJ), R. São Francisco Xavier, 524, CEP 20550-900, Rio de Janeiro, RJ, Brazil

*Corresponding author: Amanda F. do Amaral

Tel: +55 21 97625-8758

E-mail address: amaral.amanda@posgraduacao.uerj.br

ORCID 0000-0001-8339-3214

**Supplementary Material**

**Description of the Multi-Stage IFAS System (MS-IFAS)**

Commercially available fiberglass reactors were arranged sequentially as follows: Anaerobic reactor, Anoxic-1 reactor, Aerobic reactor, Sedimentation-1 tank, Anoxic-2 reactor, Re-aeration reactor, and Sedimentation-2 tank (**Figure 1**, **Table S1**). Sedimentation-1 was positioned after the aerobic reactor to enhance control over suspended biomass and to minimize dissolved oxygen (DO) transfer to the Anoxic-2 reactor. In comparison to the conventional modified Bardenpho process, the MS-IFAS system implemented in this study included an additional internal recirculation step to return sludge from Sedimentation-1 to the aerobic reactor (**Figure 1**), ensuring stable aerobic biomass concentrations.

**Table S1** Operational conditions and parameters applied to different treatment units in the MS-IFAS.

| **Treatment units** | **Volume (L)** | **Operational conditions** |
| --- | --- | --- |
| Anaerobic (Ana) | 40 | HRT=1.01 - 1.71 h |
| Anoxic-1 (Anx1) | 40 | HRT=0.64 - 1.13 h |
| Aerobic (Aer) | 240+40* | HRT=6.62 - 11.2 h; SRT=21 days |
| Anoxic-2 (Anx2) | 40 | HRT=1.01 - 1.71 h |
| Re-aeration (Re-aer) | 40 | HRT=0.76-1.28 h |
| Sedimentation-2 (Sed2) | 12 - 70 | HRT=0.38 - 1.6 h |
| Recirculation Sed1-aerobic (Sed1-Aer) | - | R=4:1 |
| Recirculation aerobic-anoxic 1 (Aer-Anx1) | - | R=1:1 |
| Recirculation Sed2-anaerobic (Sed2-Ana) | - | R=1:1 |

^SRT = sludge retention time; HRT = hydraulic retention time; R = ratio *sedimentation-1 tank.^

All recirculation streams were controlled using peristaltic pumps (Watson-Marlow, models 313 and 520R2). Sludge withdrawal from Sedimentation-1 was carried out every 21 days, and no significant losses in volume were observed due to accumulation or evaporation.

Aeration in the aerobic zone was provided by an ACQ-012 air compressor (150 L min^-1^) coupled with air curtain diffusers (Boyu), while mixing in the anoxic and anaerobic reactors was ensured by WD-2404 stepper motors (Wotiom) connected to helical stirrers (model 326006721-Consul, Brazil), operating at 80 rpm. Hydraulic retention time (HRT) values (**Table S1 and S2**) were progressively adjusted during the experimental period in response to influent flow variations to enhance chemical oxygen demand (COD) reduction efficiency and limit DO carryover to the anaerobic and anoxic compartments.

**Table S2:** Physicochemical and other parameters analyzed during the monitoring period and operation parameters used in pilot-scale multi-stage IFAS system (MS-IFAS).

|  | | | | | | **Dissolved Oxygen - DO (mg L^-1^)** | | | | | | | | | | | | | | | | | | | | | | | | | | | | | | | | | **Chemical Oxygen Demand - COD (mg L^-1^)** | | | | | | | | | | | | | | | | | | | | | | | | | | | | | | | | | | | | | **Ammoniacal Nitrogen – NH_4_^+^-N (mg L^-1^)** | | | | | | | | | | | | | | | | | | | | | | | | | | | | |
| --- | --- | --- | --- | --- | --- | --- | --- | --- | --- | --- | --- | --- | --- | --- | --- | --- | --- | --- | --- | --- | --- | --- | --- | --- | --- | --- | --- | --- | --- | --- | --- | --- | --- | --- | --- | --- | --- | --- | --- | --- | --- | --- | --- | --- | --- | --- | --- | --- | --- | --- | --- | --- | --- | --- | --- | --- | --- | --- | --- | --- | --- | --- | --- | --- | --- | --- | --- | --- | --- | --- | --- | --- | --- | --- | --- | --- | --- | --- | --- | --- | --- | --- | --- | --- | --- | --- | --- | --- | --- | --- | --- | --- | --- | --- | --- | --- | --- | --- | --- | --- | --- | --- | --- | --- |
| **Week** | | | **Inf** | | | | | | | **Ana** | | | | | **Anx1** | | | | **Aer** | | | | | | **Anx2** | | | | | **Re-aer** | | | | | **Eff** | | | | **Inf** | | | | | | **Ana** | | | | | **Anx1** | | | | **Aer** | | | | | | **Anx2** | | | | | | **Re-aer** | | | | **Eff** | | | | | | **Inf** | | | | | **Ana** | | | | **Anx1** | | | **Aer** | | | | | | **Anx2** | | | | **Re-aer** | | | | **Eff** | | |
| **1** | | | 1.03 | | | | | | | 0.04 | | | | | 0.13 | | | | 2.17 | | | | | | 0.52 | | | | | 4.13 | | | | | 4.02 | | | | 378.4 | | | | | | 349.2 | | | | | 306.7 | | | | 173.4 | | | | | | 162.6 | | | | | | 195.1 | | | | 127.6 | | | | | | 53.3 | | | | | 50.9 | | | | 26.3 | | | 2.5 | | | | | | 1.5 | | | | 1.8 | | | | 2.0 | | |
| **2** | | | 1.40 | | | | | | | 0.07 | | | | | 0.13 | | | | 2.78 | | | | | | 1.25 | | | | | 6.50 | | | | | 6.10 | | | | 380.1 | | | | | | 615.1 | | | | | 636.7 | | | | 166.7 | | | | | | 228.4 | | | | | | 231.7 | | | | 147.6 | | | | | | 52.7 | | | | | 39.1 | | | | 18.7 | | | 0.3 | | | | | | 0.2 | | | | 0.2 | | | | 0.4 | | |
| **3** | | | 0.75 | | | | | | | 0.03 | | | | | 0.21 | | | | 3.10 | | | | | | 1.28 | | | | | 6.50 | | | | | 5.36 | | | | 229.2 | | | | | | 411.7 | | | | | 209.2 | | | | 160.1 | | | | | | 183.4 | | | | | | 197.6 | | | | 146.7 | | | | | | 40.8 | | | | | 32.7 | | | | 25.5 | | | 2.3 | | | | | | 2.5 | | | | 1.3 | | | | 1.0 | | |
| **4** | | | 1.17 | | | | | | | 0.04 | | | | | 0.65 | | | | 4.37 | | | | | | 3.10 | | | | | 7.50 | | | | | 7.50 | | | | 403.7 | | | | | | 292.8 | | | | | 214.5 | | | | 119.5 | | | | | | 165.3 | | | | | | 143.7 | | | | 198.7 | | | | | | 55.1 | | | | | 36.3 | | | | 22.3 | | | 0.8 | | | | | | 0.3 | | | | 0.1 | | | | 0.0 | | |
| **5** | | | 1.30 | | | | | | | 0.26 | | | | | 0.48 | | | | 5.86 | | | | | | 2.80 | | | | | 6.40 | | | | | 6.76 | | | | 351.2 | | | | | | 310.3 | | | | | 144.5 | | | | 162.0 | | | | | | 162.0 | | | | | | 203.7 | | | | 135.3 | | | | | | 46.1 | | | | | 25.2 | | | | 12.4 | | | 0.1 | | | | | | 0.1 | | | | 0.1 | | | | 0.1 | | |
| **6** | | | 1.70 | | | | | | | 0.04 | | | | | 0.05 | | | | 3.20 | | | | | | 0.35 | | | | | 2.40 | | | | | 3.84 | | | | 304.5 | | | | | | 412.0 | | | | | 602.0 | | | | 145.3 | | | | | | 196.2 | | | | | | 127.8 | | | | 103.7 | | | | | | 50.2 | | | | | 33.3 | | | | 24.4 | | | 9.4 | | | | | | 9.1 | | | | 7.7 | | | | 7.3 | | |
| **7** | | | 1.30 | | | | | | | 0.07 | | | | | 0.13 | | | | 3.60 | | | | | | 0.20 | | | | | 0.50 | | | | | 3.22 | | | | 302.8 | | | | | | 283.7 | | | | | 359.5 | | | | 147.8 | | | | | | 242.0 | | | | | | 85.3 | | | | 70.3 | | | | | | 48.7 | | | | | 19.6 | | | | 11.4 | | | 0.1 | | | | | | 0.1 | | | | 0.7 | | | | 0.7 | | |
| **8** | | | 1.25 | | | | | | | 0.05 | | | | | 0.09 | | | | 2.54 | | | | | | 0.30 | | | | | 3.48 | | | | | 5.53 | | | | 490.7 | | | | | | 724.0 | | | | | 509.0 | | | | 187.3 | | | | | | 177.0 | | | | | | 138.2 | | | | 104.5 | | | | | | 49.8 | | | | | 34.1 | | | | 21.4 | | | 1.9 | | | | | | 1.1 | | | | 0.2 | | | | 0.1 | | |
|  | **Nitrite - NO_2_^-^-N (mg L^-1^)** | | | | | | | | | | | | | | | | | | | | | | | | | | | | | | | | | **Nitrate - NO_3_^-^-N (mg L^-1^)** | | | | | | | | | | | | | | | | | | | | | | | | | | | | | | **Total Nitrogen (mg L^-1^)** | | | | | | | | | **COD/Ammoniacal Nitrogen - C/N** | | | | | | | | | | | | | | | | | | | | | | | | | | | | | | | |
| **Week** | **Inf** | | | | | | | **Ana** | | | | **Anx1** | | | | | **Aer** | | | | **Anx2** | | | | **Re-aer** | | | | **Effl** | | | | | **Inf** | | **Ana** | | | | | **Anx1** | | | | | | **Aer** | | | | **Anx2** | | | | **Re-aer** | | | | | | **Eff** | | **Inf** | | | | | | | | **Eff** | **Inf** | | | | | **Ana** | | | | | **Anx1** | | | | **Aer** | | | | | | **Anx2** | | | | | **Re-aer** | | | | **Eff** | | | |
| **1** | 1.07 | | | | | | | 2.76 | | | | 4.67 | | | | | 3.95 | | | | | 3.43 | | | 3.82 | | | | 4.03 | | | | | 0.5 | | 12.6 | | | | | 6.0 | | | | | | 18.1 | | | | 17.8 | | | | 17.5 | | | | | | 17.2 | | 68.1 | | | | | | | | 29.5 | 7.1 | | | | | 6.7 | | | | | 11.7 | | | | 68.7 | | | | | | 106.7 | | | | | 108.0 | | | | 62.6 | | | |
| **2** | 0.19 | | | | | | | 2.81 | | | | 3.16 | | | | | 1.02 | | | | | 0.64 | | | 1.26 | | | | 1.88 | | | | | NA | | NA | | | | | NA | | | | | | NA | | | | NA | | | | NA | | | | | | NA | | NA | | | | | | | | NA | 7.2 | | | | | 15.7 | | | | | 34.0 | | | | 517.6 | | | | | | 1131.1 | | | | | 1126.7 | | | | 360.5 | | | |
| **3** | 0.14 | | | | | | | 3.1 | | | | 3.51 | | | | | 1.38 | | | | | 0.92 | | | 0.78 | | | | 0.73 | | | | | NA | | NA | | | | | NA | | | | | | NA | | | | NA | | | | NA | | | | | | NA | | NA | | | | | | | | NA | 5.6 | | | | | 12.6 | | | | | 8.2 | | | | 69.7 | | | | | | 72.6 | | | | | 149.5 | | | | 146.9 | | | |
| **4** | 0.18 | | | | | | | 2.37 | | | | 1.98 | | | | | 0.82 | | | | | 0.29 | | | 0.12 | | | | 0.06 | | | | | NA | | NA | | | | | NA | | | | | | NA | | | | NA | | | | NA | | | | | | NA | | NA | | | | | | | | NA | 7.3 | | | | | 8.06 | | | | | 9.6 | | | | 155.7 | | | | | | 649.0 | | | | | 27028.4 | | | | 19866.7 | | | |
| **5** | 0 | | | | | | | 2.33 | | | | 1.14 | | | | | 0.26 | | | | | 0 | | | 0 | | | | 0 | | | | | NA | | NA | | | | | 10.0 | | | | | | 20.4 | | | | 21.1 | | | | NA | | | | | | 20.4 | | 43.6 | | | | | | | | 24.2 | 7.6 | | | | | 12.3 | | | | | 11.7 | | | | 1132.8 | | | | | | 5842.1 | | | | | 3840.9 | | | | 4515.5 | | | |
| **6** | 0.06 | | | | | | | 0.47 | | | | 0.41 | | | | | 1.81 | | | | | 0.61 | | | 1.33 | | | | 1.54 | | | | | 0.3 | | 0.4 | | | | | 0.5 | | | | | | 4.7 | | | | 5.3 | | | | 5.9 | | | | | | 5.9 | | 55.2 | | | | | | | | 20 | 6.1 | | | | | 12.4 | | | | | 24.7 | | | | 15.48 | | | | | | 21.5 | | | | | 16.6 | | | | 14.3 | | | |
| **7** | 0.14 | | | | | | | 0.63 | | | | 0.51 | | | | | 0.89 | | | | | 0.76 | | | 2.38 | | | | 2.98 | | | | | 0.5 | | 0.4 | | | | | 1.0 | | | | | | 9.2 | | | | 9.0 | | | | 8.1 | | | | | | 7.9 | | 69.3 | | | | | | | | 19.9 | 6.2 | | | | | 14.5 | | | | | 31.5 | | | | 1926.9 | | | | | | 3965.2 | | | | | 123.5 | | | | 96.2 | | | |
| **8** | 0.1 | | | | | | | 0.02 | | | | 0.13 | | | | | 1.61 | | | | | 1.15 | | | 0.8 | | | | 0.66 | | | | | 0.4 | | 0.4 | | | | | 0.6 | | | | | | 8.0 | | | | 7.8 | | | | 8.4 | | | | | | 8.3 | | 62.1 | | | | | | | | 17.8 | 9.8 | | | | | 21.2 | | | | | 23.7 | | | | 97.5 | | | | | | 161.0 | | | | | 816.8 | | | | 859.7 | | | |
|  | | | | | **Total Suspended Solids – TSS (mg L^-1^)** | | | | | | | | | | | | | | | | | | | | | | | | | | | | | | | | | | | | | | **TSS Internal  (mg L^-1^)** | | | | | | | | | | | | | | | | **Fixed biomass (mg L^-1^)** | | | | | **COD/TSS** | | | | | | | | | | | | | | | | | | | | | | | | | | **Volumetric Organic Loading**  **Rate - VOLR**  **(kgCOD m^-^³ day^-1^)** | | | | | | | | | | | | | | |
| **Week** | | | | **Inf** | | | | | | | **Ana** | | | | | **Anx1** | | | | | | | **Aer** | | | **Anx2** | | | | | **Re-aer** | | | | | | **Eff** | | | | | **Ana** | | | | | | **Anx1** | | | | **Aer** | | | | | | **Aer** | | | | | | | **Inf** | | | | **Ana** | | | | | **Anx1** | | | | | **Aer** | | | | | **System** | | | | | **Ana** | | | | | | **Anx1** | | | | **Aer** | | | | **Anx2** | |
| **1** | | | | 65.0 | | | | | | | 58.3 | | | | | 78.3 | | | | | | | 81.7 | | | 66.7 | | | | | 36.7 | | | | | | 21.7 | | | | | 218.3 | | | | | | 240.0 | | | | 3290.0 | | | | | | 5400.0 | | | | | | | 27.4 | | | | 54.5 | | | | | 0.3 | | | | | 17.1 | | | | | 0.6 | | | | | 5.99 | | | | | | 8.3 | | | | 1.75 | | | | 4.12 | |
| **2** | | | | 73.3 | | | | | | | 260.0 | | | | | 338.3 | | | | | | | 41.7 | | | 81.7 | | | | | 90.0 | | | | | | 26.7 | | | | | 278.3 | | | | | | 305.0 | | | | 4531.7 | | | | | | 4570.0 | | | | | | | 14.7 | | | | 58.1 | | | | | 0.4 | | | | | 22.2 | | | | | 0.4 | | | | | 4.1 | | | | | | 11.07 | | | | 2.8 | | | | 3 | |
| **3** | | | | 43.3 | | | | | | | 186.7 | | | | | 60.0 | | | | | | | 51.7 | | | 65.0 | | | | | 75.0 | | | | | | 53.3 | | | | | 285.0 | | | | | | 405.0 | | | | 3001.7 | | | | | | 5090.0 | | | | | | | 9.8 | | | | 30.0 | | | | | 0.2 | | | | | 43.6 | | | | | 0.3 | | | | | 2.81 | | | | | | 7.86 | | | | 0.94 | | | | 3.05 | |
| **4** | | | | 86.7 | | | | | | | 118.3 | | | | | 100.0 | | | | | | | 53.3 | | | 0.0 | | | | | 43.3 | | | | | | 10.0 | | | | | 330.0 | | | | | | 191.7 | | | | 3970.0 | | | | | | 3390.0 | | | | | | | 11.4 | | | | 39.1 | | | | | 0.2 | | | | | 30.6 | | | | | 0.4 | | | | | 3.78 | | | | | | 4.74 | | | | 0.84 | | | | 1.94 | |
| **5** | | | | 66.7 | | | | | | | 95.0 | | | | | 90.0 | | | | | | | 51.7 | | | 48.3 | | | | | 68.3 | | | | | | 3.33 | | | | | 176.7 | | | | | | 101.7 | | | | 4060.0 | | | | | | 2520.0 | | | | | | | 14.3 | | | | 64.8 | | | | | 0.1 | | | | | 36.9 | | | | | 0.2 | | | | | 2.53 | | | | | | 4.36 | | | | 0.47 | | | | 2.27 | |
| **6** | | | | 73.3 | | | | | | | 220.0 | | | | | 380.0 | | | | | | | 51.7 | | | 93.3 | | | | | 23.3 | | | | | | 0.0 | | | | | 290.0 | | | | | | 178.3 | | | | 395.0 | | | | | | 2360.0 | | | | | | | 7.6 | | | | 49.9 | | | | | 1.4 | | | | | 21.6 | | | | | 0.2 | | | | | 2.19 | | | | | | 5.93 | | | | 1.99 | | | | 2.09 | |
| **7** | | | | 40.0 | | | | | | | 48.3 | | | | | 188.3 | | | | | | | 26.7 | | | 160.0 | | | | | 8.3 | | | | | | 10.0 | | | | | 48.3 | | | | | | 141.7 | | | | 58.3 | | | | | | 2610.0 | | | | | | | 45.1 | | | | 43.3 | | | | | 0.9 | | | | | 12.3 | | | | | 0.2 | | | | | 2.18 | | | | | | 4.08 | | | | 1.19 | | | | 2.13 | |
| **8** | | | | 100.0 | | | | | | | 550.0 | | | | | 371.7 | | | | | | | 43.3 | | | 66.7 | | | | | 35.0 | | | | | | 15.0 | | | | | 468.3 | | | | | | 333.3 | | | | 103.3 | | | | | | 2670.0 | | | | | | | 7.5 | | | | 46.9 | | | | | 1.3 | | | | | 11.8 | | | | | 0.3 | | | | | 3.53 | | | | | | 10.43 | | | | 1.68 | | | | 2.7 | |
|  | | **Conductivity (mS cm^-1^)** | | | | | | | | | | | | | | | | | | | | | | | | | | | | | | | | | | | | **pH** | | | | | | | | | | | | | | | | | | | | | | | | | | | | | | | | | | | | | **Temperature (°C)** | | | | | | | | | | | | | | | | | | | | | | | | | | | | |  |
| **Week** | | **Inf** | | | | | | | **Ana** | | | | **Anx1** | | | | | **Aer** | | | | | | **Anx2** | | | | **Re-aer** | | | | **Eff** | | | | | | **Inf** | | | | | | **Ana** | | | | | **Anx1** | | | | **Aer** | | | | **Anx2** | | | | | **Re-aer** | | | | | | **Eff** | | | | | | | **Inf** | | | **Ana** | | | | | **Anx1** | | | | **Aer** | | | | **Anx2** | | | | | **Re-aer** | | | | **Eff** | | | |  |
| **1** | | 1541 | | | | | | | 1644 | | | | 1403 | | | | | 1238 | | | | | | 1231 | | | | 1235 | | | | 124 | | | | | | 7.65 | | | | | | 7.78 | | | | | 7.54 | | | | 6.71 | | | | 6.57 | | | | | 6.71 | | | | | | 6.85 | | | | | | | NA | | | NA | | | | | NA | | | | NA | | | | NA | | | | | NA | | | | NA | | | |  |
| **2** | | 1956 | | | | | | | 165 | | | | 1252 | | | | | 1069 | | | | | | 1056 | | | | 1066 | | | | 1091 | | | | | | 7.77 | | | | | | 7.74 | | | | | 7.47 | | | | 6.71 | | | | 6.49 | | | | | 6.57 | | | | | | 6.62 | | | | | | | 28.3 | | | 27.9 | | | | | 27.3 | | | | 27.3 | | | | 27.1 | | | | | 26.9 | | | | 27.2 | | | |  |
| **3** | | 984 | | | | | | | 1049 | | | | 1046 | | | | | 946 | | | | | | 932 | | | | 93 | | | | 933 | | | | | | 7.64 | | | | | | 7.75 | | | | | 7.77 | | | | 6.76 | | | | 6.45 | | | | | 6.29 | | | | | | 6.12 | | | | | | | 32.4 | | | 30.1 | | | | | 29.1 | | | | 28.2 | | | | 28.1 | | | | | 28.1 | | | | 28.6 | | | |  |
| **4** | | 1275 | | | | | | | 1167 | | | | 1110 | | | | | 1016 | | | | | | 1011 | | | | 998 | | | | 1033 | | | | | | 7.65 | | | | | | 7.74 | | | | | 7.58 | | | | 6.19 | | | | 6.2 | | | | | 6.51 | | | | | | 6.42 | | | | | | | 25.4 | | | 24.4 | | | | | 23.8 | | | | 23.8 | | | | 23.4 | | | | | 23.3 | | | | 23.3 | | | |  |
| **5** | | 1293 | | | | | | | 1305 | | | | 1198 | | | | | 1113 | | | | | | 1113 | | | | 1148 | | | | 1167 | | | | | | 7.48 | | | | | | 7.5 | | | | | 7.3 | | | | 6.31 | | | | 5.91 | | | | | 6.01 | | | | | | 6.07 | | | | | | | 25.5 | | | 23.3 | | | | | 22.9 | | | | 22.7 | | | | 22.7 | | | | | 22.6 | | | | 22.5 | | | |  |
| **6** | | 1003 | | | | | | | 975 | | | | 928 | | | | | 849 | | | | | | 845 | | | | 845 | | | | 848 | | | | | | 7.68 | | | | | | 7.56 | | | | | 7.48 | | | | 7.35 | | | | 7.3 | | | | | 7.34 | | | | | | 7.62 | | | | | | | 24.9 | | | 23.3 | | | | | 23 | | | | 23 | | | | 23 | | | | | 23 | | | | 22.9 | | | |  |
| **7** | | 1419 | | | | | | | 1422 | | | | 1211 | | | | | 909 | | | | | | 917 | | | | 891 | | | | 906 | | | | | | 7.59 | | | | | | 7.36 | | | | | 7.22 | | | | 6.95 | | | | 6.96 | | | | | 7.01 | | | | | | 7.09 | | | | | | | 27 | | | 25.8 | | | | | 26 | | | | 26 | | | | 26 | | | | | 26 | | | | 25.9 | | | |  |
| **8** | | 1207 | | | | | | | 1131 | | | | 1014 | | | | | 777 | | | | | | 811 | | | | 816 | | | | 876 | | | | | | 7.36 | | | | | | 7.71 | | | | | 7.56 | | | | 7.22 | | | | 7.23 | | | | | 7.31 | | | | | | 7.29 | | | | | | | 31.1 | | | 30.8 | | | | | 28.7 | | | | 28.5 | | | | 28.7 | | | | | 28.7 | | | | 28.9 | | | |  |
|  | | | | | | | **Hydraulic Retention Time – HRT (h)** | | | | | | | | | | | | | | | | | | | | | | | | | | | | | | | | | | | | | | | | | | | | | | | | | **Flow rate (L min^-1^)** | | | | | | | | | | | | | | | | | | | | | | | | | | | | | | | | | | | | | | | | | | | | | | | |  |
| **Week** | | | | | | | **System** | | | | | | | **Ana** | | | | | | **Anx1** | | | | | | | **Aer** | | | | | | **Anx2** | | | | | | | **Re-aer** | | | | | | **Sed2** | | | | | | | | | | **Influent** | | | | | | | | | | | **Sed2-Ana** | | | | | | | | | | | | | **Aer-Anx1** | | | | | | | | | | | | | **Sed1-Aer** | | | | | | | | | | |  |
| **1** | | | | | | | 10.34 | | | | | | | 1.01 | | | | | | 0.64 | | | | | | | 6.62 | | | | | | 1.01 | | | | | | | 0.76 | | | | | | 0.3 | | | | | | | | | | 0.44 | | | | | | | | | | | 0.22 | | | | | | | | | | | | | 0.38 | | | | | | | | | | | | | 0.46 | | | | | | | | | | |  |
| **2** | | | | | | | 13.63 | | | | | | | 1.33 | | | | | | 0.83 | | | | | | | 8.73 | | | | | | 1.33 | | | | | | | 1 | | | | | | 0.4 | | | | | | | | | | 0.30 | | | | | | | | | | | 0.20 | | | | | | | | | | | | | 0.30 | | | | | | | | | | | | | 0.45 | | | | | | | | | | |  |
| **3** | | | | | | | 12.89 | | | | | | | 1.26 | | | | | | 0.81 | | | | | | | 8.24 | | | | | | 1.26 | | | | | | | 0.94 | | | | | | 0.38 | | | | | | | | | | 0.34 | | | | | | | | | | | 0.19 | | | | | | | | | | | | | 0.29 | | | | | | | | | | | | | 0.45 | | | | | | | | | | |  |
| **4** | | | | | | | 15.16 | | | | | | | 1.48 | | | | | | 0.94 | | | | | | | 9.7 | | | | | | 1.48 | | | | | | | 1.11 | | | | | | 0.44 | | | | | | | | | | 0.26 | | | | | | | | | | | 0.19 | | | | | | | | | | | | | 0.26 | | | | | | | | | | | | | 0.45 | | | | | | | | | | |  |
| **5** | | | | | | | 17.54 | | | | | | | 1.71 | | | | | | 1.13 | | | | | | | 11.2 | | | | | | 1.71 | | | | | | | 1.28 | | | | | | 0.51 | | | | | | | | | | 0.20 | | | | | | | | | | | 0.19 | | | | | | | | | | | | | 0.20 | | | | | | | | | | | | | 0.45 | | | | | | | | | | |  |
| **6** | | | | | | | 17.11 | | | | | | | 1.67 | | | | | | 1.11 | | | | | | | 10.92 | | | | | | 1.67 | | | | | | | 1.25 | | | | | | 0.5 | | | | | | | | | | 0.20 | | | | | | | | | | | 0.20 | | | | | | | | | | | | | 0.20 | | | | | | | | | | | | | 0.45 | | | | | | | | | | |  |
| **7** | | | | | | | 17.11 | | | | | | | 1.67 | | | | | | 1.11 | | | | | | | 10.92 | | | | | | 1.67 | | | | | | | 1.25 | | | | | | 0.5 | | | | | | | | | | 0.20 | | | | | | | | | | | 0.20 | | | | | | | | | | | | | 0.20 | | | | | | | | | | | | | 0.45 | | | | | | | | | | |  |
| **8** | | | | | | | 17.11 | | | | | | | 1.67 | | | | | | 1.11 | | | | | | | 10.92 | | | | | | 1.67 | | | | | | | 1.25 | | | | | | 0.5 | | | | | | | | | | 0.20 | | | | | | | | | | | 0.20 | | | | | | | | | | | | | 0.20 | | | | | | | | | | | | | 0.45 | | | | | | | | | | |  |

Legend: **Ana**: Anaerobic reactor; **Anx1**: Anoxic-1 reactor; **Aer**: Aerobic reactor; **Anx2**: Anoxic-2 reactor; **Re-aer**: Re-aeration reactor; **Sed1**: Sedimentation-1 tank; **Sed2**: Sedimentation-2 tank (effluent); **Inf**: Influent; **Eff**: Effluent; **Sed2-Ana**: Recirculation sedimentation-2-anaerobic; **Aer-Anx1**: Recirculation aerobic-anoxic 1; **Sed1-Aer**: Recirculation Sed1-aerobic; **NA:** parameter not analyzed on the respective sampling day.

**Physicochemical analysis**

Temperature and DO were measured using a luminescent dissolved oxygen probe (Hach LDO10130), while pH and electrical conductivity were assessed with a multiparameter water quality probe (Horiba U-52, Tokyo, Japan). Ammoniacal nitrogen, nitrite, nitrate, total nitrogen, COD, total suspended solids (TSS), and the complete solids series were analyzed by the methodologies described in the Standard Methods (APHA et al., 2017).

The fixed biomass adhered to the carriers, expressed as total attached solids (TAS) and volatile attached solids (VAS), was quantified using a protocol developed in-house, which combines ultrasonic dispersion with manual agitation (Silva et al., 2020).

Three carriers removed from the reactor were transferred to an Erlenmeyer flask containing 50 mL of deionized water and subjected to ultrasonication (40 kHz) for 90 minutes. During the process, every 5 minutes the flasks were manually shaken for approximately 3 seconds. The resulting liquid fraction was analyzed for suspended solids according to Method 2540D (APHA et al., 2017). After extraction, the supports were dried in an oven at 105°C for 24 hours, cooled, and weighed (MMext). They were then immersed in 50 mL of 1 mol L^-1^ NaOH solution in an Erlenmeyer flask and kept under heating for 30 minutes, with temperature reduction during the last 15 minutes. Flasks were shaken every 10 minutes during heating. After treatment, the supports were thoroughly washed with tap and deionized water, dried in an oven at 105°C for 24 hours, cooled, and weighed again (MMwash).

In this study, total attached solids (TAS) were determined from the sum of the total suspended solids (TSS) concentration converted to the total volume of the vial from which the aliquot was taken, with the residual mass attached to the media after the extraction process according to **Equation S1**.

$$TAS =MA+TSS (S1)$$

Where: *TAS* is the total attached solids; *MA* is the mass of attached solids; and *TSS* is the total suspended solids.

The mass of solids attached to the carriers (MA) was determined by the difference between the mass of the carriers after the extraction process and the mass after washing, according to **Equation S2**.

$$MA =MMext-MMwash (S2)$$

Where: MMext is the mass of carriers after extraction; and MMwash = mass of carriers after washing.

The extraction efficiency (Eeff) was calculated from the ratio of the mass of extracted solids to the total mass of solids on the carriers according to **Equation S3**.

$$Eeff =100 \frac{TSS}{TSS+TAS} (S3)$$

**Chromatographic Analysis**

For sample concentration, solid phase extraction (SPE) was applied according to the method described in the literature (Pugajeva et al., 2017) with modifications. Before extraction, the samples were filtered through a glass fiber membrane (0.7 µm). A volume of 250 mL from each sample was then acidified to pH 3.0 using 3 M HCl, followed by the addition of 25 µL of 0.5 M EDTA solution to enhance extraction efficiency. SPE was performed using Supel Select HLB® cartridges (200 mg/6 mL) in a manifold system (Phenomenex).

Cartridge conditioning was carried out under vacuum at an approximate flow rate of 2 mL min^-1^ (drip mode) with sequential washes of 5 mL methanol and 5 mL ultrapure water. The acidified samples passed through the cartridge at a controlled flow rate of 3 mL min^-1^. After sample percolation, the cartridge was dried under vacuum for 30 minutes. For the elution of the analytes, 6 mL of a methanol: dichloromethane (70:30) solution, was used, followed by solvent evaporation applying nitrogen gas. The final extract was reconstituted with 1.25 mL of a methanol: water (1:1) solution, resulting in a 200-fold enrichment.

BPA and PBS were determined with an ACQUITY® ultra-performance liquid chromatography system coupled to a triple quadrupole mass spectrometer Xevo TQD® Waters (UPLC-MS/MS). Chromatographic separation was performed using an Acquity UPLC® BEH C18 column (1.7 µm, 2.1 mm × 50 mm, Waters, Milford, MA, USA) maintained at 50 °C. The mobile phase consisted of water (phase A) and methanol (phase B), both containing 0.01% ammonium hydroxide. The flow rate was set at 0.4 mL min^-3^ and the injection volume was 5 µL. The total running time was 6 min, following a gradient elution program: the initial condition of 90% Phase A was maintained for 4 min, then reduced to 1% for 5 min, and then returned to the initial composition, where it remained until the end of the analysis.

The mass spectrometer was operated in negative electrospray ionization (ESI) mode with multiple reaction monitoring (MRM). The capillary voltage was set at 3.2 kV, while the source and desolvation temperatures were maintained at 150 °C and 600 °C, respectively. Nitrogen was used as both the cone gas (150 L h^-1^) and desolvation gas (1,100 L h^-1^), while argon was used as the collision gas in the MS/MS detector at a flow rate of 0.15 mL min^-1^. Specific MS/MS parameters are detailed in **Table S3**. Data processing was performed using MassLynx v4.1 software (Waters, Milford, MA, USA).

**Table S3: Optimized parameters of the MS/MS detector for the studied analytes.**

| **Analytes** | **Ionization Mode (ESI)** | **Precursor (m/z)** | **Quantification** | | | **Confirmation** | | |
| --- | --- | --- | --- | --- | --- | --- | --- | --- |
|  |  |  | **Product (m/z)** | **CE**  **(V)** | **Cone (V)** | **Product (m/z)** | **CE**  **(V)** | **Cone (V)** |
| Bisphenol A | - | 227.0 | 212.0 | 18 | 45 | 133.0 | 25 | 45 |
| Bisphenol S | - | 249.0 | 108.0 | 25 | 35 | 159.0 | 25 | 35 |
| Bisphenol Ad_16_ | - | 241.3 | 223.3 | 19 | 45 | 142.3 | 25 | 45 |

^CE: Collision energy; ESI: Electrospray Ionization.^

Validation of the analytical method included evaluation of linearity, linear range, limit of detection (LOD), limit of quantification (LOQ), accuracy (recovery), precision, and matrix effects (ICH, 2005).

Calibration curves were generated using analytical standards to encompass the expected concentration range of each analyte in environmental samples. These curves were constructed based on the ratio of analyte to surrogate peak area versus analyte concentration, ensuring compliance with a coefficient of determination (R² > 0.99) and validation by the lack-of-fit ANOVA test. The accuracy of the method, as assessed by repeatability (n=6) and reproducibility across days, met the acceptance criterion of a relative standard deviation (RSD) of less than 15% (**Table S4**).

LOD and LOQ were determined based on the 3-fold and 10-fold standard deviation of the mean blank signal (n=6), respectively, divided by the slope of the corresponding calibration curve.

**Table S4: UPLC-MS/MS analytical quality control parameters for BPA and BPS.**

| **Parameter** | **BPA** | | **BPS** | |
| --- | --- | --- | --- | --- |
|  | **Influent** | **Effluent** | **Influent** | **Effluent** |
| Linear range (µg L^-1^) | 10-200 | 1-150 | 1-150 | 1-150 |
| n | 6 | 8 | 7 | 8 |
| Limit of detection (µg L^-1^) | 0.50 | 0.50 | 0.50 | 0.50 |
| Limit of quantification (µg L^-1^) | 1.00 | 1.00 | 1.00 | 1.00 |
| Method detection limit (µg L^-1^) | 0.0025 | 0.0025 | 0.0025 | 0.0025 |
| Method quantification limit (ng L^-1^) | 0.005 | 0.005 | 0.005 | 0.005 |
| Matrix Effect (%) | 104 | 93 | 272 | 973 |
| RSD_1_ (%) | 2.3 | 6.3 | 1.7 | 11.5 |
| RSD_2_ (%) | 6.8 | 7.1 | 3.2 | 2.4 |
| Equation | y=-0.497 + 0.04898x | y=-0.0272 + 0.054714x | y=0.0161 + 0.036642x | y=-0.0230 + 0.010233x |
| R² | 0.9905 | 0.9984 | 0.9996 | 0.9904 |
| F_calc_ value | 2.36 | 1.130 | 2.540 | 0.920 |
| p-value | 0.112 | 0.387 | 0.078 | 0.504 |

*^RSD1^* ^- relative standard deviation of repeatability;^ *^RSD2^* ^relative standard deviation of inter-day^

Due to the challenges associated with sample collection, analytical automation, and the complexity of the sample matrix, an alternative quantification strategy was used. Samples from the anaerobic and anoxic-1 compartments were quantified using the influent calibration curve, while samples from the aerobic compartment were quantified using the effluent calibration curve. The matrix effect (ME) was evaluated according to **Equation S4**.

$ME \left( \% \right)=\left( slopematrix\div slopeIS \right)\times100$ *(S4)*

Where: *slopematrix* is the calibration curves matrix prepared with residual water; and *slopeIS* is the calibration curves prepared with ultrapure water.

The recovery of SPE extraction was evaluated at two concentration levels for each analyte: low (0.1 µg L^-1^) and high (0.2 µg L^-1^). The recovery results for each analyte are shown in **Figure S1**. For BPA and BPS at 0.2 µg L^-1^, recoveries were within the internationally recommended range of 70%-120%. However, at the lower concentration level (0.1 µg L^-1^), recoveries slightly exceeded 120%.

**Figure S1 -** SPE extraction recovery (%) of BPA and BPS, starting with two concentrations: 0.1 µg L^-1^ and 0.2 µg L^-1^.

Note: The dotted lines represent the acceptable recovery range from 50% to 120%.

Due to the complexity of the matrix studied, a broader recovery range from 50% to 120% was adopted to ensure compliance with the accuracy criteria of the method, which allows deviations of up to ±15%.

**Calculation Procedures**

*Hydraulic Retention Time (HRT)*

The hydraulic retention time was obtained from reactional volume of each reactor to the flow rate, is shown in **Equation S5**.

| $HRT=\frac{V}{Q}$ | (S5) |
| --- | --- |

Where: *V* is the reactional volume in L; and *Q* is the flow rate in L h^-1^.

*Volumetric Organic Loading Rate (VOLR)*

To obtain the volumetric organic loading rate in kg COD m^-3^ day^-1^, **Equation S6** was used.

| $VOLR=\frac{Q\cdot S}{V}$ | (S6) |
| --- | --- |

Where: *Q* is the flow rate in m^3^ d^-1^; *S* is the Substrate concentration in the influent in Kg COD m^-3^; and *V* is the reactional volume in m^3^.

*Removal efficiency*

The removal efficiency of a given parameter was defined using **Equation S7**.

| $\eta=\left( 1-\frac{Ce}{Ci} \right)\cdot100$ | (S7) |
| --- | --- |

Where: *η* is the removal efficiency in %, *Ci* is the influent concentration and *Ce* is the effluent concentration.

**Tables**

**Table S5 -** Composition of the culture media used in the YES assay. Routledge and Sumpter (1996).

A. Minimal medium (per 1 L of double-distilled water, pH 7.1).

| **Component** | **Amount** |
| --- | --- |
| KH_2_PO_4_ | 13.61 g |
| (NH_4_)_2_SO_4_ | 1.98 g |
| KOH pellets | 4.2 g |
| MgSO_4_ | 0.2 g |
| Fe₂(SO_4_)_3_ solution | 1 mL (40 mg in 50 mL H₂O) |
| L-Leucine | 50 mg |
| L-Histidine | 50 mg |
| Adenine | 50 mg |
| L-Arginine-HCl | 20 mg |
| L-Methionine | 20 mg |
| L-Tyrosine | 30 mg |
| L-Isoleucine | 30 mg |
| L-Lysine-HCl | 30 mg |
| L-Phenylalanine | 25 mg |
| L-Glutamic acid | 100 mg |
| L-Valine | 150 mg |
| L-Serine | 375 mg |

B. Vitamin solution (per 200 mL of double-distilled water).

| **Component** | **Amount** |
| --- | --- |
| Thiamine | 8 mg |
| Pyridoxine | 8 mg |
| Pantothenic acid | 8 mg |
| Inositol | 40 mg |
| Biotin solution | 20 mL (2 mg in 100 mL H₂O) |

^Note: The vitamin solution was filter-sterilized (0.2 µm) and stored at 4 °C.^

C. Stock solutions.

| **Stock solution** | **Concentration** |
| --- | --- |
| D-(+)-Glucose | 20% (w/v) |
| L-Aspartic acid | 4 mg mL^-1^ |
| L-Threonine | 24 mg mL^-1^ |
| Copper (II) sulfate | 20 mM |
| CPRG | 10 mg L^-1^ |

D. Growth medium preparation.

| **Component added to 45 mL minimal medium** | **Volume added** |
| --- | --- |
| Glucose solution | 5.0 mL |
| L-Aspartic acid solution | 1.25 mL |
| Vitamin solution | 0.50 mL |
| L-Threonine solution | 0.40 mL |
| Copper (II) sulfate solution | 125 µL |

E. Assay medium preparation.

| **Component** | **Amount** |
| --- | --- |
| Growth medium | 50 mL |
| CPRG solution | 0.5 mL |
| Yeast culture (OD_640_ = 1.0) | 2.0 mL |

**Table S6 –** Results of the Shapiro-Wilk normality test for each bisphenol and comparison of removal efficiencies among reactors (Ana, Aer, and Anx1) and the overall system.

| **Parameter** | **Normality Test**  **Shapiro-Wilk (p-value)** | **Test** | **p-value** | **Sign. diff.** |
| --- | --- | --- | --- | --- |
|  |  |  |  |  |
| BPA removal (%) | 0.000 | Kruskal-Wallis | 0.000 | Yes |
| BPS removal (%) | 0.000 | Kruskal-Wallis | 0.000 | Yes |

**Table S7 -** Dunn's Test of Multiple Comparisons for BPA and BPS removal.

| **Comparison** | **p-value**  **BPA** | **p-value**  **BPS** |
| --- | --- | --- |
| Aer – Ana | 0.037 | 0.020 |
| Aer – Anx1 | 0.036 | 0.033 |
| Ana – Anx1 | 0.925 | 0.778 |
| Aer - System | 0.107 | 0.174 |
| Ana - System | 0.000 | 0.000 |
| Anx1 – System | 0.000 | 0.000 |

**Aer**: Aerobic; **Anx1**: Anoxic 1; **Ana**: Anaerobic.

**Table S8 -** Bartlett's sphericity test.

| **Bartlett's sphericity test** |
| --- |
| p-value 0.000 |

**Table S9 -** Calculated eigenvalues for the first five PCs (%) and variance.

| **Eigenvalues** | **PC1** | **PC2** | **PC3** | **PC4** | **PC5** |
| --- | --- | --- | --- | --- | --- |
| Variance | 7.243 | 1.453 | 1.055 | 0.764 | 0.607 |
| % of Variance | 60.357 | 12.105 | 8.795 | 6.365 | 5.059 |
| Cumulative % of Variance | 60.357 | 72.462 | 81.257 | 87.622 | 92.681 |

**Table S10 -** Contributions (in %), correlations (r), and p-values for each correlation between qualitative variables on PCs 1 and 2.

| **Variables** | **PC1** | | | **PC2** | | |
| --- | --- | --- | --- | --- | --- | --- |
|  | **Cont. (%)** | **r** | **p-value** | **Cont. (%)** | **r** | **p-value** |
| DO | 12.002 | 0.932 | 0.000 | 1.362 | 0.141 |  |
| HRT | 11.599 | 0.917 | 0.000 | 4.653 | 0.260 |  |
| pH | 10.468 | -0.871 | 0.000 | 0.247 | -0.060 |  |
| Temperature | 2.313 | -0.409 |  | 46.184 | 0.819 | 0.000 |
| VOLR | 6.353 | -0.678 | 0.002 | 3.037 | -0.210 |  |
| C/N ratio | 5.504 | 0.631 | 0.005 | 0.820 | 0.109 |  |
| COD reduction | 3.364 | 0.513 | 0.029 | 1.059 | -0.124 |  |
| Ammoniacal nitrogen removal | 12.447 | 0.949 | 0.000 | 4.335 | 0.251 |  |
| BPA removal | 12.333 | 0.945 | 0.000 | 1.764 | 0.160 |  |
| BPS removal | 11.918 | 0.929 | 0.000 | 0.072 | 0.032 |  |
| BPA influent |  | -0.547 | 0.019 |  | 0.525 | 0.025 |
| BPS influent |  | -0.727 | 0.000 |  | 0.504 | 0.033 |

**BPA**: Bisphenol A; **BPS**: Bisphenol S **DO:** Dissolved oxygen; **HRT**: Hydraulic Retention Time; **VOLR**: Volumetric organic loading rate; **C/N**: COD/Ammoniacal nitrogen; **COD**: Chemical oxygen demand; **PC1, PC2 and PC3:** Principal Components 1, 2 and 3, respectively; **Cont. (%):** Contributions in % of the original variables to the principal components in PCA (the 10 first); r = linear correlations between the original variables and the principal components.

**Table S11 -** Homoscedasticity, normality test, and correlation tests with BPA and BPS removal for different variables used.

| **Correlation** | **Homoscedasticity**  **Breusch Pagan**  **(p-** **value)** | **Normality**  **Shapiro - Wilk**  **(p-** **value)** | | **Correlation test** | | | |
| --- | --- | --- | --- | --- | --- | --- | --- |
|  |  |  |  | **Test** | **p-value** | **Coeff.** | **Strength*** |
| **Anaerobic, Anoxic-1, and Aerobic reactors** | | | | | | | |
| BPA remov vs COD reduct | 0.081 | 0.313 | Pearson | | **0.012** | 0.535 | moderate |
| BPA vs Namon removals | 0.514 | 0.399 | Pearson | | **0.000** | 0.916 | very strong |
| BPA remov vs C/N ratio | 0.037 | 0.000 | Spearman | | **0.000** | 0.741 | strong |
| BPA remov vs VOLR | 0.150 | 0.046 | Spearman | | **0.000** | -0.764 | strong |
| BPA remov vs HRT | 0.270 | 0.114 | Pearson | | **0.000** | 0.906 | very strong |
| BPA remov vs BPA influ | 0.799 | 0.016 | Spearman | | **0.048** | -0.439 | moderate |
| BPA remov vs BPS influ | 0.588 | 0.016 | Spearman | | **0.000** | -0.697 | strong |
| BPA remov vs DO | 0.095 | 0.302 | Pearson | | **0.000** | 0.847 | very strong |
| BPA remov vs pH | 0.011 | 0.428 | Spearman | | **0.000** | -0.855 | very strong |
| BPA remov vs Temp | 0.231 | 0.028 | Spearman | | 0.167 | -0.340 | weak |
| BPA vs BPS removals | 0.031 | 0.046 | Spearman | | **0.000** | 0.874 | very strong |
| BPS remov vs COD reduct | 0.014 | 0.382 | Spearman | | **0.018** | 0.517 | moderate |
| BPS vs Namon removals | 0.057 | 0.991 | Pearson | | **0.000** | 0.931 | very strong |
| BPS remov vs C/N ratio | 0.063 | 0.000 | Spearman | | **0.002** | 0.635 | strong |
| BPS remov vs VOLR | 0.129 | 0.037 | Spearman | | **0.000** | -0.756 | strong |
| BPS remov vs HRT | 0.196 | 0.104 | Pearson | | **0.000** | 0.788 | strong |
| BPS remov vs BPA influ | 0.942 | 0.007 | Spearman | | **0.027** | -0.486 | moderate |
| BPS remov vs BPS influ | 0.632 | 0.511 | Pearson | | **0.002** | -0.646 | strong |
| BPS remov vs DO | 0.196 | 0.313 | Pearson | | **0.000** | 0.750 | strong |
| BPS remov vs pH | 0.096 | 0.037 | Spearman | | **0.000** | -0.867 | very strong |
| BPS remov vs Temp | 0.893 | 0.071 | Pearson | | 0.107 | -0.393 | weak |
| **Anaerobic** | | | | | | | |
| BPA remov vs COD reduct | 0.143 | 0.301 | Pearson | | 0.174 | 0.578 | moderate |
| BPA vs Namon removals | 0.144 | 0.412 | Pearson | | **0.009** | 0.881 | very strong |
| BPA remov vs C/N ratio | 0.204 | 0.408 | Pearson | | 0.974 | 0.016 | very weak |
| BPA remov vs VOLR | 0.146 | 0.567 | Pearson | | 0.197 | -0.554 | moderate |
| BPA remov vs HRT | 0.193 | 0.956 | Pearson | | 0.128 | 0.632 | strong |
| BPA remov vs BPA influ | 0.373 | 0.060 | Pearson | | 0.805 | 0.116 | very weak |
| BPA remov vs BPS influ | 0.728 | 0.125 | Pearson | | **0.045** | -0.764 | strong |
| BPA remov vs DO | 0.144 | 0.043 | Spearman | | 0.077 | 0.704 | strong |
| BPA remov vs pH | 0.798 | 0.040 | Spearman | | **0.034** | -0.821 | very strong |
| BPA remov vs Temp | 0.202 | 0.378 | Pearson | | 0.128 | -0.692 | strong |
| BPA remov vs BPS remov | 0.253 | 0.358 | Pearson | | **0.001** | 0.943 | very strong |
| BPS remov vs COD reduc | 0.020 | 0.634 | Spearman | | 0.556 | 0.286 | weak |
| BPS vs Nammon removals | 0.274 | 0.144 | Pearson | | **0.000** | 0.973 | very strong |
| BPS remov vs C/N ratio | 0.455 | 0.176 | Pearson | | 0.593 | 0.248 | weak |
| BPS remov vs VOLR | 0.033 | 0.921 | Spearman | | 0.065 | -0.726 | strong |
| BPS remov vs HRT | 0.494 | 0.673 | Pearson | | **0.019** | 0.835 | very strong |
| BPS remov vs BPA influ | 0.166 | 0.099 | Pearson | | 0.992 | 0.004 | very weak |
| BPS remov vs BPS influ | 0.217 | 0.811 | Pearson | | **0.005** | -0.903 | very strong |
| BPS remov vs DO | 0.113 | 0.123 | Pearson | | 0.215 | 0.536 | moderate |
| BPS remov vs pH | 0.720 | 0.312 | Pearson | | **0.004** | -0.910 | very strong |
| BPS remov vs Temp | 0.728 | 0.522 | Pearson | | 0.139 | -0.677 | strong |
| **Anoxic-1** | | | | | | | |
| BPA remov vs COD reduct | 0.169 | 0.791 | Pearson | | 0.663 | -0.203 | weak |
| BPS vs Nammon removals | 0.332 | 0.600 | Pearson | | 0.939 | 0.036 | very weak |
| BPA remov vs C/N ratio | 0.146 | 0.552 | Pearson | | 0.158 | 0.595 | moderate |
| BPA remov vs VOLR | 0.365 | 0.066 | Pearson | | 0.360 | -0.411 | moderate |
| BPA remov vs HRT | 0.228 | 0.304 | Pearson | | **0.000** | 0.993 | very strong |
| BPA remov vs BPA influ | 0.577 | 0.056 | Pearson | | 0.358 | -0.412 | moderate |
| BPA remov vs BPS influ | 0.617 | 0.751 | Pearson | | **0.002** | -0.929 | very strong |
| BPA remov vs DO | 0.664 | 0.138 | Pearson | | 0.873 | 0.075 | very weak |
| BPA remov vs pH | 0.343 | 0.467 | Pearson | | 0.140 | -0.616 | strong |
| BPA remov vs Temp | 0.758 | 0.353 | Pearson | | 0.383 | -0.440 | moderate |
| BPA remov ves BPS remov | 0.565 | 0.960 | Pearson | | 0.864 | 0.080 | very weak |
| BPS remov vs COD reduct | 0.553 | 0.340 | Pearson | | 0.899 | 0.060 | very weak |
| BPS vs Nammon removals | 0.355 | 0.746 | Pearson | | **0.004** | 0.914 | very strong |
| BPS remov vs C/N ratio | 0.393 | 0.542 | Pearson | | 0.807 | -0.115 | very weak |
| BPS remov vs VOLR | 0.976 | 0.138 | Pearson | | 0.259 | -0.495 | moderate |
| BPS remov vs HRT | 0.413 | 0.032 | Spearman | | 0.691 | 0.185 | very weak |
| BPS remov vs BPA influ | 0.799 | 0.005 | Spearman | | 0.236 | -0.536 | moderate |
| BPS remov vs BPS influ | 0.338 | 0.156 | Pearson | | 0.575 | -0.259 | weak |
| BPS remov vs DO | 0.721 | 0.123 | Pearson | | 0.364 | 0.408 | moderate |
| BPS remov vs pH | 0.756 | 0.444 | Pearson | | 0.100 | -0.670 | strong |
| BPS remov vs Temp | 0.627 | 0.272 | Pearson | | 0.080 | -0.759 | strong |
| **Aerobic** | | | | | | | |
| BPA remov vs COD reduct | 0.586 | 0.487 | Pearson | | 0.805 | -0.115 | very weak |
| BPA vs Namon removals | 0.170 | 0.110 | Pearson | | 0.533 | 0.287 | weak |
| BPA remov vs C/N ratio | 0.076 | 0.396 | Pearson | | 0.203 | 0.548 | moderate |
| BPA remov vs VOLR | 0.880 | 0.747 | Pearson | | 0.817 | 0.108 | very weak |
| BPA remov vs HRT | 0.311 | 0.047 | Spearman | | 0.937 | 0.037 | very weak |
| BPA remov vs BPA influ | 0.143 | 0.753 | Pearson | | 0.505 | 0.306 | weak |
| BPA remov vs BPS influ | 0.668 | 0.405 | Pearson | | 0.979 | 0.013 | very weak |
| BPA remov vs DO | 0.458 | 0.171 | Pearson | | 0.669 | -0.199 | very weak |
| BPA remov vs pH | 0.428 | 0.875 | Pearson | | 0.466 | 0.332 | weak |
| BPA remov vs Temp | 0.378 | 0.629 | Pearson | | 0.437 | 0.396 | weak |
| BPA remov vs BPS remov | 0.247 | 0.143 | Pearson | | **0.028** | 0.808 | very strong |
| BPS remov vs COD reduct | 0.707 | 0.524 | Pearson | | 0.937 | -0.037 | very weak |
| BPS vs Nammon removals | 0.772 | 0.007 | Spearman | | 1 | 0 | very weak |
| BPS remov vs C/N ratio | 0.106 | 0.203 | Pearson | | 0.427 | 0.360 | weak |
| BPS remov vs VOLR | 0.684 | 0.660 | Pearson | | 0.698 | 0.181 | very weak |
| BPS remov vs HRT | 0.515 | 0.060 | Pearson | | 0.923 | -0.045 | very weak |
| BPS remov vs BPA influ | 0.794 | 0.135 | Pearson | | 0.857 | 0.085 | very weak |
| BPS remov vs BPS influ | 0.079 | 0.784 | Pearson | | 0.566 | 0.246 | very weak |
| BPS remov vs DO | 0.877 | 0.299 | Pearson | | 0.551 | -0.275 | very weak |
| BPS remov vs pH | 0.854 | 0.331 | Pearson | | 0.229 | 0.522 | moderate |
| BPS remov vs Temp | 0.623 | 0.621 | Pearson | | 0.445 | 0.389 | weak |

**BPA**: Bisphenol-A; **BPS**: Bisphenol-S; **COD**: Chemical oxygen demand; **C/N**: COD/Ammonia; **VOLR**: Volumetric Organic loading rate; **HRT**: Hydraulic Retention Time; **DO:** Dissolved oxygen. Pearson test applied for those data sets that follow normal distribution and Sperman test for those that do not follow normal distribution. In **bold,** p-values indicating statistical significance. *(Evans, 1996)

**Table S12 -** Shapiro-Wilk normality test results for each reactor with bisphenols (BPA and BPS) removal data and comparison of their efficiencies.

| **Reactor** | **Comparison** | **Normality Test**  **Shapiro-Wilk (p-** **value)** | **Test** | **p-value** | **Sign. diff.** |
| --- | --- | --- | --- | --- | --- |
| Anaerobic | Removal *vs* Substance | 0.734 | Indep. Samples T-test | 0.840 | No |
| Anoxic-1 | Removal *vs* Substance | 0.976 | Indep. Samples T-test | 0.116 | No |
| Aerobic | Removal *vs* Substance | 0.002 | Mann-Whitney Test | 0.011 | Yes |
| Aerobic | Removal *vs* Phase | 0.002 | Mann-Whitney Test | 0.662 | No |

**Table S13 -** Physicochemical properties of the BPA and BPS.

| **Parameter** | **BPA** | | **BPS** |
| --- | --- | --- | --- |
| Chemical formula ^a,b^ | | C_15_H_16_O_2_ | C_12_H_10_O_4_S |
| CAS number ^a,b^ | | 80-05-7 | 80-09-1 |
| Log K_ow_^a^ | | 3.32 | 1.65 |
| Molecular weight (g mol^-1^)^a,b^ | | 228.29 | 250.27 |
| Melting point (°C)^a^ | | 155 | 176 |
| Henry Constant (atm m^3^ mol^-1^)^a^ | | 1.26 x 10^-7^ | 3.55 x 10^-11^ |
| Chemical structure^b^ | | 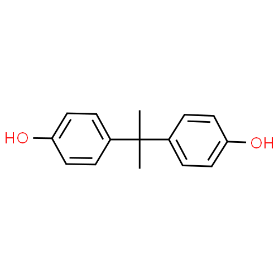 | 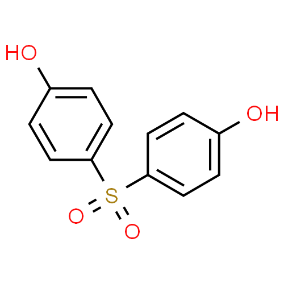 |

a EPA: available in: <https://comptox.epa.gov/dashboard/>. b Chemspider: available in: < http://www.chemspider.com/ >

**FIGURES**

**Figure S2 -** Dose‐response curve for the positive control E2 (LOD = 0.01 ng L^‐1^ and LOQ = 0.02 ng L^‐1^).

**References**

APHA, AWWA, WEF, 2017. Standard Methods for the Examination of Water and Wastewater, 23rd ed. American Public Health Association, Washington DC.

Evans, J.D., 1996. Straightforward Statistics for the Behavioral Sciences. Brooks/Cole Pub Co, Pacific Grove, CA.

ICH, 2005. Validation of Analytical Procedures: Text and Methodology Q2 (R1). Int. Conf. Harmon.

Pugajeva, I., Rusko, J., Perkons, I., Lundanes, E., Bartkevics, V., 2017. Determination of pharmaceutical residues in wastewater using high performance liquid chromatography coupled to quadrupole-orbitrap mass spectrometry. J Pharm Biomed Anal 133, 64–74. https://doi.org/10.1016/j.jpba.2016.11.008

Routledge, E.J., Sumpter, J.P., 1996. Estrogenic activity of surfactants and some of their degradation products assessed using a recombinant yeast screen. Environ Toxicol Chem 15, 241–248. https://doi.org/10.1002/etc.5620150303

Silva, A.S.A. da, Castro, R.A. de, Martins, S.B., Alvim, R.D., Salomão, A.L. de S., Marques, M., 2020. Extração de biomassa aderida ao meio-suporte de um reator de leito móvel com biofilme: agitação mecânica e ultrassom (Extraction of biomass attached to a support media in a moving bed biofilm reactor: mechanical agitation and ultrasound, in Portuguese). Eng Sanit Ambient 25, 901–908. https://doi.org/10.1590/s1413-4152202020180113
